# Clinical Review, and Hospital Reports

**Published:** 1842-07-01

**Authors:** 


### Clinical Review.

---

### GUY'S HOSPITAL REPORTS.

Guy's Hospital Reports. No. XIV. April, 1842. Edited by George H. Barlow, M.A. & M.D. Candidate of the Royal College of Physicians, Assistant Physician to Guy's Hospital, and Physician to the Surrey Dispensary; and James P. Babington, M.A. Trin. Coll. Cam., Member of the Royal College of Physicians.

The Number of the Reports before us contains the following Articles:—Cases illustrative of the Diagnosis of Disease of the Kidney; by G. H. Barlow, M.A. and M.D.—Medico-Legal Report of a Case of Infanticide, with additional Remarks on the Fœtal Lungs; by Alfred S. Taylor—Observations on Pelvic Tumors obstructing Parturition, with Cases; by John C. W. Lever, F.S.S.—Observations on the Digestive Solution of the Œsophagus, and on the distinct Properties of the Two Ends of the Stomach; by T. Wilkinson King—Two Cases of Injury to the Head, followed by Symptoms of Compression produced respectively by Extravasation of Blood and Formation of Pus, relieved by Operation; by Edward Cock—Observations on Urinary Concretions and Deposits, with an Account of the Calculi in the Museum of Guy's Hospital; by Golding Bird, A.M. M.D. F.L.S.—On the Location of Pulmonary Phthisis, and its relation to Diagnosis, &c. &c.; by H. Marshall Hughes, M.D.—On the Proceeding to be adopted in a Case of Injured Intestine from a Blow upon a Hernial Sac; by C. Aston Key—Case of Irideremia, or absence of Iris, with Observations; by John Frederick France—Case of Enormously-distended Gall-Bladder: communicated by B. G. Babington, M.D. F.R.S.

### I. Cases illustrative of the Diagnosis of Disease of the Kidney.

Dr. Barlow observes, that these cases have reference chiefly to the diagnosis between disease of the kidney, and that of the bladder, or vertebral column, or lumbar muscles: The points which he thinks they tend chiefly to illustrate, are these:—

1. That there is a certain symptom connected with irritation of the kidney, and which, although not confined to it alone, is not necessarily connected with disease of those structures whose affections are most likely to be confounded with those of the kidneys. So that when this symptom is absent, we may eliminate, and therefore disembarrass ourselves of the consideration of all such affections of the kidneys as would necessarily be attended by irritation of that organ: and when this symptom is present, we are furnished with a reason for assigning the seat of the disease to the kidney, in preference to several adjacent structures. The symptom in question is sickness, or, rather, irritability of the stomach.

2. And further, that there is a certain symptom, or rather set of symptoms, dependent upon the non-depuration of the blood by the kidney, whether this non-depuration be the result of mechanical obstruction to the flow of the urine, of a diminution in its quantity, or of a depraved condition of that secretion in which its most important ingredients are wanting—cerebral disorder of a peculiar character.

The cases which follow, are too numerous and too circumstantial to admit of our fully noticing them. We must content ourselves with alluding to the more prominent.

The *first* case is one of *Hæmaturia, of obscure origin, found to proceed from a Fungus in the Bladder.* There was a difference of opinion on the nature of the case. There was no sickness.

CASE 2. *Pains in the Loins, of doubtful origin.—Sickness.—Tuberculated Kidney.*

Eleanor W. aged 65, April 22nd, 1835. Ten years before she had suffered from what seemed a nephritic attack. Three years after that, she became suddenly hemiplegic, losing the use of her left side and the sight of her right eye: from this she recovered, but for six months she had been gradually getting ill. At that time she was somewhat emaciated, and had an expression of great anxiety in her countenance. She complained of a constant aching pain in the region of the right kidney; and was at times suddenly seized with a lancinating pain in the same situation; the violence of which was such, that she frequently alarmed the whole neighbourhood with her screams, the pain extending down the anterior and inner surface of the right thigh. Her urine was natural in quantity, and quality. Her stomach was very irritable, and she vomited frequently. Her pulse and tongue were natural.

She had occasional headaches, though this symptom was never very severe; and though very irritable, she was never delirious; neither had she *stupor, stertor, drowsiness,* or *coma.* Morphia gave most relief, but she grew worse, and died comatose on the 11th July, 1837.

*Dissection.*—Upon removing the calvaria, which was unusually thick, a considerable quantity of fluid escaped. The arachnoid was then seen to be much thickened, particularly upon the anterior and superior surfaces of the anterior lobes of the brain. This thickening was produced by both recent and cellular adventitious membrane. There was considerable serous effusion between the arachnoid and pia mater. The brain was very firm, and the cineritious substance darker than natural. There was a cell, of about the size of a pea, in the right corpus striatum. There was some serous effusion under the dura mater of the medulla spinalis; but the contents of the spinal canal were otherwise healthy. The left kidney was large: the right was externally nodulated; and, on making an incision through it, it was found to be thickly studded with tubera, varying in size from that of a pea to that of a cob-nut. These tubera were mostly of a dirty-yellow colour, and firm consistence; but one or two appeared to be in a state of softening.

CASE 3 is one of *Pain in the Loins, with Exacerbations occurring in Paroxysms.—Sickness.—Irritating Calculi in the Right Kidney.*

CASE 4 is one of *Obscure Pains in the Loins, with Sickness; disappearing upon the passage of a Calculus.*

CASE 5. *Great irritability of Stomach.—Death with Heart-disease, at the end of a year and a half.—A small Calculus in the Left Kidney.*

"Lucy C——, was admitted under my care, at the Surrey Dispensary, in the Summer of 1840, with evident symptoms of disease of the right side of the heart, accompanied with most obstinate irritability of the stomach; so that for several days she could retain scarcely any thing besides effervescing draughts. She obtained partial relief: but again became my patient in the Autumn of the following year; when, in addition to the heart-symptoms, which were of increased severity, she complained of pain in the left lumbar region, which was at times intense, and acccompanied by sickness. She died on the 8th of December, 1841.

The body was examined the following day, by Mr. Nettlefold, and Mr. J. Miles. The disease of the heart, lungs, and liver, that had been diagnosed during life, having been found, I was asked if I was satisfied. I replied, 'No; unless some cause of irritation were found in the left kidney.' After making two or three incisions into that organ, a small sharp-pointed calculus, apparently composed of lithic acid, was found in its upper part near the line of union of the tubular and cortical structures."

Dr. Barlow observes :—In the last four cases, there was present the symptom first mentioned as dependent upon irritation of the kidney; whilst the cerebral disturbance, noticed secondly, as resulting from impaired function of the kidneys, was absent: and accordingly, in the three fatal cases, sufficient cause of irritation was found in these organs, whilst their general structure was so far healthy as to afford no impediment to the function of secretion. In the two cases which follow, the cerebral disorder, as well as the irritability of stomach, was observed during life; and, accordingly, we shall find causes of irritation of the kidney co-existing with causes of obstruction of the function of that organ.

Of two cases related in illustration, we select one.

*Case.*—Miss H———, aged between 35 and 40, was, in the Winter of 1838-9, under my care, with fever of rather low type. The most distressing symptom, throughout her illness, was, inability to retain any nourishment on her stomach; the exhaustion produced by which was such as to excite considerable alarm. During that time, and subsequently, her friends remarked a degree of apathy, which did not naturally belong to her. In September 1840, I again saw her; when she was suffering from severe headache, attended with stupor: from this she in great measure recovered; but there still remained an apathy to all that was going on around her, which was hardly overcome by a strong sense of duty. On the 27th of April last she again consulted me, with an erythematous swelling of the legs, which was attributed to amenorrhœa. She got better, and went to Brighton: but, after having been there a few days, was seized with violent pain in the right hypochondrium and right lumbar region, attended with sickness. This was in some manner relieved, as I learnt, by fomentation and aperients. She was brought home; and on the 17th of May I again saw her. The pain had by that time returned with great severity, and was accompanied with considerable tenderness. She did not complain of much sickness, but of an apprehension that taking any thing would produce vomiting. Her tongue was furred, with very red edges: urine scanty, and dark: no œdematous swelling. She was cupped, and treated with calomel and opium, with small doses of tartarized antimony, and moderate purgatives. She was on the following day much relieved; but I then discovered that her urine, which was dark and scanty, was highly albuminous. She continued slightly to improve for about a fortnight; though she scarcely once passed twenty-four hours without vomiting, the matter ejected from her stomach being always green and acid. At the end of that time, a copious crop of purpura appeared, both on the trunk and extremities: these continued to increase for about three days; after which they became stationary, and then began to fade. Her urine, which was dark throughout, assumed a brighter colour whilst the purpura was at its height. The sickness again became urgent, and signs of sinking came on. She was at no time delirious, but became more and more apathetic. She died on the 15th of June.

*Dissection.* The head was not opened.

There was a considerable quantity of bloody serum in the right pleural sac, and a very little in the left. There was a mass of pulmonary apoplexy, occupying nearly the whole extent of the upper lobe of the left lung; and the same was observed, though to a less extent, in the lower lobe on the same side: the right lung was healthy, with the exception of some patches of pulmonary apoplexy. The heart was small, and firmly contracted, all its cavities being empty; except the right auricle, which was moderately filled with fluid blood.

The liver was pale: near its acute edge, and for some distance backwards, it was hard; but near the obtuse margin it became soft, though of the same colour as anteriorly. The spleen was large and firm.

Both kidneys were of great size, and very coarse in their structure. The enlargement was owing, apparently, to hypertrophy of the cortical portion; the tubular structure being much encroached upon, and the pelvis nearly obliterated. The right ureter was, externally, of the size of a man's thumb, from the pelvis of the kidney to within an inch of the bladder: below this, it was of natural dimensions. The enlarged portion, when cut into, appeared to consist of thickened and condensed cellular membrane, which encroached so much upon the duct as to render it nearly impervious. The mucous membrane of the bladder was, in parts, much injected. There were some large patches of ecchymosis on various parts of the external surface of the alimentary canal, and the mucous membrane of the corresponding parts was much softened.

Dr. Barlow supposes, from the thickened state of the coats of the ureter, that this patient at one time passed a calculus.

Dr. Barlow proceeds:—There are several affections of the kidney which do not necessarily give rise to irritation of that organ, and in which the sickness *may* not be present to aid our diagnosis: of these, the principal are the granular degeneration, suppuration, and perhaps some forms of adventitious or malignant deposit.

In the first of these, the disease depending, in the first place, probably upon congestion, and afterwards upon a chronic change in the structure of the organ, irritation is not a necessary concomitant; although the disease may, and no doubt frequently does, result from irritation giving rise to chronic inflammation: accordingly, we find that sickness is not always present, although it is sometimes a most troublesome symptom.

In suppuration, we should generally have irritation giving rise to sickness, in the first instance; and probably, at a later period, obstructed function, with its concomitant changes in the urine; and cerebral disorder, with probably, at some time, the presence of pus in the urine.

With regard to malignant disease, it is very probable that this deposit taking place, as has been remarked by Dr. Bright, in the cellular membrane connecting the firm parts of various structures, the parts of the organ in which this deposit takes place may be, as it were, pushed aside, without suffering any mechanical violence or functional disturbance; at the same time, it is also probable that, in the progress of the distention to which this organ is subjected, some slight laceration, or irregular pressure, giving rise to irritation, will sooner or later occur; and, accordingly, some sudden invasion of sickness almost uniformly happens, before such deposit in the kidney has produced tumor of any considerable size. We should also in this case, probably, have some degree of hæmaturia, especially if the disease be of a fungoid character, the most common form of malignant deposit in the kidney: but this is not a necessary consequence.

Three cases follow.

CASE 7 is interesting.

*Hæmaturia—Obstinate Sickness—Fungoid Kidney.*

Dr. Golding Bird communicates the case. Ann Harrington, aged 16, admitted May 17, 1833. Her symptoms then were occasional hæmaturia, with lumbar pains, resembling those of impending menstruation, which function had never occurred. For this she took acetate of lead, with decoction of pareira; under the use of which the blood disappeared from the urine; and after an interval of a week or two, during which she continued tolerably well, sickness at stomach appeared: this insidiously and gradually increased, from July 13, 1833, to March 1834, with only occasional intervals of freedom: the hæmaturia returned repeatedly during these periods; and extreme agony often occurred, from a plug of coagulum obstructing the urethra. By the aid of effervescing medicine and small doses of opium, the occasional administration of hydrocyanic acid and soda-water, &c. &c., the vomiting was frequently relieved, but never disappeared. On the 16th of August, the urine, which for some months had been loaded with albumen, presented a very curious appearance, being free from blood, of a fine amber colour, and gelatinising, on cooling, like so much calf's-foot jelly, and assuming the shape of the containing vessel. This gelatinous urine subsequently became red, from the admixture of blood. Dr. Addison, at this time, regarded the case as one of fungoid disease of the kidney.

This state of the urine, with almost continual sickness and rapid emaciation, continued until April 1834; when she left the hospital, and died at Walworth a few weeks afterwards. Both kidneys were converted into masses of fungus-hæmatodes; the calices of both were dilated, and contained some hard concretions of coagulated albumen.

Case 8 is one of *Tumor in the Right Hypochondrium.—Temporary Irritation of the Stomach.—No apparent Change in the Urine.—Cerebriform Disease of the Kidney.*

Case 9 is one of *Scrofulous Tumors in the Peritoneum, giving rise to a Tumor in the Right Hypochondriac and Iliac Regions, supposed to be a Tumor of the Right Kidney.*

W. V. aged 5, a scrofulous looking boy, April 3, 1840. At thirteen months old he had had ague, which lasted for three or four months. At the age of two years and a half he had scarlatina. Three months ago an enlargement was observed in the right iliac fossa, and soon afterwards one above the pelvis; at which parts there were two glandular enlargements to be felt. His abdomen enlarged very rapidly for the first month after the appearance of the tumors, and more slowly during the two following months. His mother had died of an enlargement of the liver, said to be malignant, and which appeared about two months before his birth.

His abdomen was much distended, and there was a large lobulated tumor occupying the right iliac fossa and inguinal region, and extending upwards and to the left, so as to occupy the greater portion of the umbilical and pelvic regions: there appeared, also, to be a portion of intestine crossing it anteriorly, towards its lower portion. He was somewhat emaciated; but his general health appeared in other respects, but little affected: he was cheerful, and tolerably active, till towards evening, when he used to complain of fatigue and pain in the hypogastrium. There were some glandular enlargements about the neck. His appetite was natural and bowels regular, and *he had no sickness.* He used at times to complain of much thirst: urine seemed to be natural: tongue rather furred, and dry. He was ordered small doses of iodine, with iodide of potassium. Afterwards he took liquor potassæ, and in the course of some months he died.

*Dissection.*—On opening the abdomen, there was a large tumor formed between the layers of the right meso-colon, of an oval form, the long axis about twelve or fourteen inches, the short axis about nine inches, weighing about fifteen pounds, consisting of tuberculous matter, for the most part solid, but portions in various stages of softening. There were also two tumors of the same character between the layers of the mesentery; the larger of the two about half the size of the largest; the other a little smaller. The liver contained a number of tuberculous deposits, varying in size from that of a small bean to that of a large hen's egg.

Dr. Barlow winds up by observing:—"I would remark, that I by no means intend to assert that the irritability of stomach noticed in many of the preceding cases is pathognomonic of disease of the kidney: for, I am of opinion, that if we hope to find pathognomonic signs of disease in general, our search will be fruitless, and our diagnosis erroneous; but merely, that in cases where doubts arise as to whether any disease is to be referred to the kidney or some neighbouring organ, the absence or presence of sickness will go far to decide the question; and further, that in cases which we not unfrequently meet with—of which Case 4 is an instance—where the prominent symptom is distressing and obstinate sickness without any assignable cause, especial attention should be directed to the kidneys and every means used to determine the state of those organs.

The same observations will apply very nearly to the second class of symptoms of which I have been speaking, namely, the cerebral disorder: for we, as yet, want sufficient evidence to prove that similar derangement of the functions of the brain may not be produced by other causes. At the same time, I am fully convinced of its importance as an aid to diagnosis, and believe that it is one by which the really-accurate observer will be rarely misled."

### II. Medico-Legal Report of a Case of Infanticide; with additional Remarks on the Fœtal Lungs. By Alfred S. Taylor.

Mr. Taylor sets out with the narration of a pretty clear case of infanticide where, partly owing to the state of the law, partly to the ingenuity of counsel, and partly to the humanity of the jury, the prisoner was acquitted of the crime of murder, and convicted only of concealment of the birth.

On this peg Mr. Taylor hangs some valuable observations to which we shall, as briefly as possible, allude.

### *Disinclination of Juries to convict for Infanticide.*

Mr. Taylor observes that most trials for child-murder end in the escape of the prisoner. She is acquitted of the murder, in opposition to the strongest evidence against her, and found guilty of concealment of birth; so that no other punishment is inflicted than that to which a female would be sentenced who had been secretly delivered of a child that had died from natural causes, and the body of which she had afterwards concealed. But can the former serious crime be placed in comparison with a trivial offence of this description? If not, there is something manifestly wrong, in finding a prisoner guilty of an offence, for the sake of punishing her for a crime of which she has been acquitted. The only explanation of this anomaly in our criminal jurisprudence, is, that juries regard the law as too severe for many cases of this description, since there is no alternative between an acquittal and a capital conviction. While we admit that there are cases of infanticide in which capital punishment would perhaps be too severe, we must also allow, that other cases of murder are too severely punished; or that two years' imprisonment for a minor offence, which necessarily accompanies child-murder, is an insufficient penalty. Allowing that, in the instance before us, the child had lived a week before it was strangled, what would have been the verdict of the jury? They would probably have pronounced the prisoner guilty, although the medical proofs of life and the cause of death could not have been stronger than they were in the case of this child. And yet, so far as the repression of crime is concerned, we cannot see that there is less criminality in destroying a child which has lived only an hour, than in destroying one which has lived a week. At present, then, in this country, the effect of capital punishment being attached to the crime, in all cases, is to prevent conviction in the large majority of them. Whatever the force of medical evidence may be, juries appear to consider that the acquittal of a prisoner charged with child-murder is the lesser evil;—that society will suffer less by her liberation, than the cause of justice could gain by her execution. It is unfortunate that there is not some intermediate punishment, as in the French law, which is in this respect much superior to ours; and it is to be regretted, that, by certain constrained interpretations of the law, as to the proofs of life and live-birth, the science of medical jurisprudence is made to bear the onus of these acquittals.

*The Hydrostatic Test.*

Mr. Taylor relates several cases and experiments with the view of determining the value of this.

We pass over these and content ourselves with stating his conclusions.

1. *The Test of Ploucquet.*

This refers to the ratio of the weight of the lungs to the weight of the body. Mr. Taylor observes that " it is clear, from the cases reported in this Paper, that the test is not even capable of furnishing corroborative proof, and that it would be unsafe to rely upon it. I subjoin a Table of the Weights of the Lungs and Bodies, in the twelve cases of mature children reported; separating those which had respired from those which had not.

*Before Respiration.*

| Weight of | the body. | Lungs. | Ratio. |
|---|---|---|---|
| 1. | 57,000 gr. | 694 gr. | 1 : 82 |
| 2. | 62,660 | 683 | 1 : 91 |
| 3. | 34,540 | 630 | 1 : 54 |
| 4. | 47,170 | 703 | 1 : 67 |
| 5. | 51,890 | 744 | 1 : 70 |
| 6. | 29,460 | 520 | 1 : 57 |
| 7. | 29,966 | 666 | 1 : 45 |
| 8. | 47,025 | 658 | 1 : 71 |
| 9. | 39,370 | 550 | 1 : 71 |

The second and third cases on the Table shew that there is no sort of constant relation between the weight of the lungs and that of the body; for while the body in No. 2 weighed nearly twice as much as in No. 3, the lungs in the respective subjects differed in weight only by fifty-three grains. Thus, then, it does not follow, as it has often been stated, that when the body is below the average weight the lungs will also be below the average. There are many facts on record which bear out this view; but the two cases referred to, prove that, in practice, an inference of this kind must be cautiously employed. The ratios, it will be seen, differ so widely from each other, as to render Ploucquet's test a very uncertain guide in infanticidal investigations. In a Paper published in a late Number of the Edinburgh Medical and Surgical Journal, No. 148, Dr. Guy has come to a similar conclusion, from an examination of cases, derived partly from his own experience, and partly from that of others.

*After Respiration.*

| Weight of | the body. | Lungs. | Ratio. |
|---|---|---|---|
| 1. | 56,160 gr. | 1000 gr. | 1 : 56 |
| 2. | 34,125 | 861 | 1 : 39 |
| 3. | 41,788 | 920 | 1 : 45 |

The comparison of these ratios, obtained from lungs after respiration with those obtained before respiration, will shew that Ploucquet's test is not fitted to determine, in an unknown case, whether a child has breathed or not."

2. *Absolute Weight of the Lungs.*

The average weight before respiration, derived from the nine cases reported in the Table, is 649 grains. It is of importance, in making an estimate of this kind, to be certain that the child is at or near maturity; and it would be better, in all reports of cases relating to infanticide, if, instead of the bare assertion that the child was mature, the reporter would describe its general characters, so that every one might have the opportunity of forming a judgment on the subject.

The average weight of the lungs after respiration, derived from three cases in the Table, is 927 grains; but in making an estimate of this kind, much will depend upon the degree to which respiration has been carried. In Mr. Coales's case, where the child was probably killed soon after birth, the lungs weighed 1000 grains. In Mr. French's case (Case 7), where the child had lived eight or nine days, the lungs weighed only 861 grains. In the first case, respiration had been perfectly performed; in the second, imperfectly. This increase in weight after birth is commonly ascribed to the altered course of the blood under the establishment of the respiratory process; and to the fact, that more blood circulates through the lungs after, than before respiration. This view appears to be borne out. Mr. Taylor argues the question, and shews that the balance of evidence and of probability is on the side of increase of weight in the lung being derived from respiration.

3. *Artificial Inflation.*

In a former paper, three conclusions were derived from many experiments, viz.

1. That, in artificially-inflated lungs, the air might be forced out by compression, when the pressure was applied, not to the entire, but to the divided lung. It was stated, that this experiment had succeeded in all cases, even where the lung had been violently inflated after removal from the thorax; though in this case it was a difficult process, and required several repeated compressions. 2dly. That the air might be forced out of lungs which had imperfectly respired; and that as these did not differ in weight from the fœtal standard, there were no means of distinguishing artificial inflation from imperfect respiration, in new-born children. 3dly. That where respiration had been perfectly established, the air could not be forced out by compression, without entirely destroying the structure of the organs.

Mr. Taylor now details the results of further observations.

1. One lung was artificially inflated, when removed from the thorax, in Cases 2, 3, 5, and 6; and in all of them it will be seen, that although the inflation was carried to different degrees, the air was ultimately forced out by pressure. In Cases 2 and 3, the mere pressure between the fingers sufficed to cause several portions of lung to sink. In Cases 5 and 6, there was very great difficulty in expelling the air, even by repeated pressure in a folded cloth; and the structure of the lung was bruised, although it could not be said to be destroyed.

Mr. Taylor goes on to observe:—

" In all these instances, however, the lung was inflated externally to the chest. The real force of the objection clearly consists in the degree of inflation which we can give to the lungs, *in situ*, of a still-born child, where the air is propelled for the purpose of resuscitation. The difficulty of inflating the lungs in a new-born child is too well known to require here to be adverted to: the greater the violence used, the less likely is the air to pass into these organs; but it rather finds its way, through the œsophagus, into the stomach and bowels. In Case 1, only about one-thirtieth of the structure of the lungs had received air by inflation in the usual way. In Case 3, inflation was repeatedly resorted to, but no part of the lungs had received a trace of air: it passed entirely into the abdomen. In Case 5, attempts were made by the accoucheur, for upwards of half an hour, to inflate the lungs; but, on examination, not a particle of air had penetrated into them. In Case 6, inflation was also resorted to, but no air was found in the lungs. In Case 10, inflation of a small portion of air only entered the lungs, and this was easily forced out by compression. These observations seem to me to establish, that there is considerable difficulty in inflating the lungs of a new-born child: and if this be admitted with respect to a medical practitioner, *à fortiori* it must stand with regard to a woman secretly delivered of a child, the only form in which the objection, on the ground of artificial inflation, can be practically contemplated. But, admitting that the lungs may be thus inflated, these and other observations establish, that the air may be expelled by compression, and much more readily than where the inflation is performed, as it commonly is, externally to the thorax. Any attempt at inflation, made under the design of resuscitating a child, would, it appears to me, be easily distinguished from perfect respiration, by a practitioner acquainted with the subject. It is not pretended that there are any means by which he can distinguish lungs so inflated, from those which have imperfectly or feebly respired. It must also be remembered, in discussing this objection, that the lungs of a still-born child are not inflated under the design of causing them to resemble respired lungs in their physical characters, and to acquire great buoyancy in water; although, from the views that have been promulgated on this subject, it would seem that the only object of inflation was, not to resuscitate it, but to make its lungs resemble those of a child which had died after having fully breathed. In allowing that an accused party is entitled to every benefit arising from a medical doubt, we must not outrage all probability, and admit that a woman recently delivered can succeed in accomplishing what cannot be done by a practised accoucheur; namely, in filling every part of the structure of the lungs with air. A woman trying this experiment is necessarily ignorant of the method of performing it; and may therefore employ great violence, in which case she is still less likely to succeed."

We apprehend that this is about the last thing women will be likely to attempt, or, if they attempt it, to succeed in. A woman, indeed, must be a very learned fool to dream of such a thing.

2. The air may be forced out of lungs that have breathed imperfectly. This conclusion is clearly confirmed by the result of Case 4, a case of imperfect respiration. Here, however, very great pressure was required: still the effect was the same as if the lungs had been artificially inflated.

3. "Air cannot be forced by pressure out of lungs that have fully breathed. None of the cases which I met with admitted of this experiment being tried; but the result obtained by Mr. Coales corroborates the view above taken; and so also does Case 7, observed by Mr. French; yet here it might be fairly objected, that respiration was imperfect; and the inference ought rather to be, that, in some states of imperfect respiration, the air resisted expulsion by compression. In Case 15, observed by Dr. Geoghegan, although the lungs here were well filled, most probably by respiration, yet compression caused the greater number of pieces to sink, without entirely destroying their structure. In Case 14, that of an immature child, the lungs were well filled with air by respiration; and this was expelled on very moderate compression, so as to cause them to sink.

Taking the whole of these results, it does appear that there is too great uncertainty to allow of the application of this test of compression, to the extent to which previous experiments would have justified us in applying it."

After some farther remarks, Mr. Taylor proceeds:—

"There are two cases which might give rise to some doubt on the source of the air contained in the lungs of a new-born child:—

1. Where, in a child that has not breathed, the lungs are disproportionately heavy, weighing 900 or 1,000 grains, and they have been artificially inflated in the attempt to resuscitate it. Unless, in this case, the air were expelled by compression, an inference might be hastily drawn, that the child had probably breathed after birth. The error could only be removed by circumstantial evidence; which, however, is generally sufficient to remove a speculative objection of this kind. But unless the fœtal lungs were highly congested, diseased, or of extraordinary size, it is not likely that they would weigh so much as is here supposed. This kind of doubtful case might always be suspected to exist where, with considerable absolute weight, the lungs contain very little air.

Let us, however, consider what would be the practical bearing on the question of child-murder, supposing the case not to be cleared up by any of the methods above suggested. 1st. The fact of respiration would not be clearly proved, because the great absolute weight of the lungs, without their being fully permeated with air, amounts to nothing. 2dly. Although the proof of respiration might not be made out, this would not shew that the child was born dead; for we know that a child may live many hours, and yet no evidence of life may be derived from an examination of the lungs. 3dly. Admitting that there was proof of the child having lived after birth, whether there were evidence of respiration or not, the cause of death would have still to be made out: and unless this be clearly traced to the wilful and malicious conduct of the prisoner—proofs of which are not likely to be derived from the body of a child whose lungs she has inflated—she must be acquitted. Thus, then, it is difficult to understand how, in the hands of one who has attended to the subject of infanticide—and no others ought to be allowed to give medical evidence—this objection, on the ground of inflation, can lead to any difficulty whatever in practice.

2. We will now take the converse objection. A child may live and breathe, and its lungs weigh much under the average of respired lungs, *i. e.* about 700 grains. In a case like this, unless the air resisted expulsion by compression, an opposite mistake might be made, and we should pronounce a child that had really breathed and survived birth to have been still-born and had its lungs artificially inflated. This might happen in numerous cases of imperfect respiration after birth, did we not know that the sinking of the lungs, whether containing air or not, and whether this air be expelled by compression or not, does not prove that the child was born dead. It can only shew, under the most favourable circumstances, that it has either not respired, or respired imperfectly. The sinking of the lungs may take place in a child that has survived birth and has really been murdered; but, in such a case, there might be no proofs of life; and therefore a person really guilty of a crime must be discharged for want of sufficient medical evidence to convict. This, however, could no more justify the entire abandonment of medical evidence in such cases, than it could of general evidence; because this, like that which is purely medical, is but too often insufficient to bring home guilt to the really guilty. The objection, then, on the ground of artificial inflation, is more speculative than real. Admitting that there is no positive criterion to distinguish this condition from respiration, it is difficult to conceive a case in which it could be sustained; and if sustained, it never could lead, in the hands of proper witnesses, to the inculpation of the innocent: unfortunately for society, it would only add another loop-hole to the many that, through the necessary forms of law, now exist, for the escape of the really guilty."

### 4. *Signs of Maturity in New-born Children.*

It has been generally considered, on the authority of Chaussier, that, in a mature child, the opening for the umbilical cord corresponds to the centre of the length of the body. But the cases reported by Mr. Taylor shew, that, in mature children, the umbilicus is from a quarter to half an inch below the centre.

5. *Signs of Live Birth.*

Among these, we find enumerated the presence of a circle of redness at the junction of the umbilical cord with the skin of the abdomen. In the former Paper, the fallacy of this criterion was established, by the report of a case wherein this line of redness was discovered, although the child was born dead. In five other cases of still-born children, the circle of redness was found. On the other hand, it has not been found where the child has been born alive and survived its birth. The existence of a line of redness in this situation cannot, therefore, be regarded as a sign of live birth.

The contraction of the ductus arteriosus has been usually considered a positive evidence of live birth, although it strictly depends on the degree of respiration established. In Cases 5 and 8, where the children were born dead, Dr. Geoghegan remarked a slight contraction about the centre of the vessel. This is a point which requires to be still further investigated.

### III. Observations on Pelvic Tumors obstructing Parturition. With Cases. By John C. W. Lever, F.S.S.

After a rapid historical sketch, Mr. Lever observes:—Pelvic tumors take their origin from various parts and tissues of the pelvis; and in considering them, it will be advisable to arrange them into two classes: 1st. Those which implicate the pelvis itself, or those organs and structures concerned in the birth of the child: and, 2dly, Those tumors which belong to or implicate the parts in the neighbourhood of the birth-passages. The first division will comprehend, *A.* tumors of the bony pelvis and its ligaments; and, *B.* tumors of the uterus and vagina, which, by their size or structure, impede the progress of a natural labour. The second division will include those diseases of the ovaries, Fallopian tubes, rectum, bladder, cellular tissue, as well as those varieties of pelvic hernia which may offer an obstruction to the course of natural parturition.

1. *Tumors of the Bony Pelvis and its Ligaments.*

There are two varieties of tumor which originate in the bones of the pelvis; viz. exostosis, and osteosarcoma.

Exostoses take their origin from either the internal or external plate of the bone; while through their whole structure, and throughout their course, they preserve the normal texture of osseous matter: or the tumor may be harder than true bone, although possessing the integral, chemical, and microscopical characters of bone. Osteosarcoma is a mixed substance, partly osseous and partly fungous, more commonly proceeding from an articulation or a ligamentous structure than from a bone.

The most common situation for the commencement of pelvic exostoses is the last bone of the sacrum; they have also been found at the junction of the last lumbar vertebra with the sacrum. Occasionally, they are found growing from the pubes or ischia; but examples of exostoses growing from these bones are more rare than cases of this tumor springing from the sacrum. The situation of the tumor will have a material influence in the production of symptoms by which their presence may be suspected, particularly the pressure which their contiguity to important viscera, vessels, and nerves, enables them to exercise; but in most instances, their existence has not been dreamt of, until the period of utero-gestation has been completed, and the progress of the labour is found to be obstructed by a foreign body occupying the pelvic cavity.

The diagnosis of these tumors is by no means easy: their firm attachment to the bone itself, and their great hardness, are the two principal signs by which they are to be distinguished. Projection of the promontory of the sacrum, caused by rickets and exostosis of the sacrum, may be confounded. The state of the pelvis in other respects, and the general aspect and constitution of the patient furnish, perhaps, the principal indications.

The formation of exostosis is frequently to be traced to some external cause, as a blow, kick, fall, &c.; but there appears to be some constitutional predispotion to osseous deposit, to account for their development. Patients who have suffered from rheumatism, or those in whom the gouty diathesis exists, seem predisposed to the formation of exostosis.

The size, form, and seat of the exostosis must exercise a considerable influence upon the prospect of recovery to the mother, and safety to the child, when these tumors are complicated with pregnancy. Cases are recorded, where the pelvic cavity was completely filled with a tumor of this kind; whilst others are published, and specimens are preserved in some Museums, in which the bony tumor is so small, that if the head of the fœtus had been of normal dimensions, no great or serious impediment to its delivery could have taken place. In form these tumors are generally more or less round or conical; but if the shape of an exostosis be long, and if by its length it encroaches upon the pelvic cavity, it will render the labour more serious. The situation of the exostosis is of especial moment, for the difficulty and risk of labour is much greater when exostoses are situated in the pelvic cavity; and, besides, there is more danger when they coarct the brim of the pelvis, than when they encroach upon the outlet. Although these tumors are not generally discovered until the patient is in labour, yet a vaginal examination, made either to ascertain the condition of the uterus itself, or the causes of various symptoms, may lead the practitioner to the detection of the tumor before the patient is pregnant, or before utero-gestation was advanced too far to allow of its being terminated artificially.

After detailing a case, Mr. Lever thus touches on the treatment. " If," he says, " a tumor be discovered in a female who is pregnant, the most careful examination must be instituted, to ascertain its seat, its dimensions, and the difficulties its form may create: and after these have been satisfactorily made out, the practitioner must then determine whether he can safely permit his patient to go to the full period of gestation, and trust her delivery to the natural efforts, or to his assistance by means of the forceps, or whether the circumstances will warrant him in inducing premature labour or abortion.

If the exostosis be small, and especially if it be attached to one of the bones forming the outlet of the pelvis, the practitioner should give the patient a fair chance of delivery by the natural efforts, particularly if the pains are vigorous, and the fœtal cranium is not firmly ossified, but compressible, as in the case related by Danyau, in his Lectures. Burns quotes a case from Campbell, where, from pelvic exostosis, the left parietal bone was so greatly sunk as to cause the eye to protrude: and Dr. Rigby relates, that Professor Otto, of Breslau, mentions the case of a woman who had pelvic exostosis, being the mother of four children, in each of whom a small portion of the cranium was depressed, and not ossified. But if the surgeon determine to allow a full trial of the natural efforts, he must carefully watch his patient, lest her strength become exhausted, or lest rupture of the uterus take place. If the conjugate diameter of the pelvis will allow the introduction of more than three fingers, the forceps may be applied; and amongst the circumstances rendering their employment necessary, pelvic exostosis is mentioned by Madame Boivin, in her celebrated " Memorial." If this instrument have been employed ineffectually, or if the conjugate diameter of the pelvis measures less than the breadth of three fingers, craniotomy must be had recourse to. Some writers have recommended the operation of turning to be performed; but although version may be accomplished, the difficulties arising in the delivery of the head will not be lessened by the alteration of the position of the child: and although more extractive force can be used, yet the force necessary to effect the delivery may not only cause the death of the child, but also occasion that of the mother; as in the case of Van Döverin, alluded to by Naëgle. Symphysotomy, recommended by some writers, appears not to have been followed by success in the hands of those who have practised it; although Michell asserts, that after its performance the forceps may be applied, where previous attempts to introduce them have failed. Gardien states, that in exostosis of the base of the sacrum impeding the descent of the fœtus, symphysotomy is the only remedy whereby a living child can be delivered. If the exostosis be not discovered until the completion of utero-gestation ; or if it be of large size, as in the extraordinary case of Dr. Haber, of Carlsruhe; or if it be so located as to prevent the introduction of the forceps; or if it be impossible to extract the child, even after the cranial contents have been evacuated; the only mode of delivery is by performing the Cæsarian section. Ruleau, Leydig, M'Kibbin, (Edinburgh Medical and Surgical Journal, Vol. XXXV.), Spitzbarth, and Siebold, have performed the Cæsarian section in labours complicated with pelvic exostosis. But if we have the opportunity of ascertaining that such a tumor exists, which will prevent the delivery of the patient by the natural efforts or by the employment of the forceps or vectis, or if its dimensions are such that perforation must be performed, or even the Cæsarian section itself may be necessary, it is most assuredly our duty to induce artificial delivery; the period of pregnacy, at which the operation is to be performed, depending altogether on the size and situation of the pelvic obstruction."

Of *Osteosarcoma*, we need only observe that—fixed pain generally marks the inflammation which attends the onset of these tumors. Their diagnosis is very difficult: when one exists, it may be mistaken for the fœtal head; but its origin from some part of the bones of the pelvis, and its consistence, are the two grand distinctive marks that assist in the diagnosis. This latter feature must also enable us to distinguish between those tumors and pelvic exostosis; the latter being hard and unyielding throughout; the former being hard in some places, soft in others; in some places permitting compression, in others resisting it. Its form, also, is occasionally irregular.

*Ossicula from Fracture of the Pelvis* should be alluded to. They occasionally project much into its interior.

### TUMORS OF THE UTERUS AND VAGINA.

Mr. Lever treats of uterine tumors under these heads :—*a*. Induration of the os and cervix, the result of chronic inflammation. *b*. Abscess of the neck of the womb. *c*. Elongation of the anterior lip of the os uteri. Specific tumors, including, *d*. Hard or fibrous tumor. And *e*. Polypi and malignant tumors; embracing, *f*. Carcinoma, and Cauliflower Excrescence.

#### *a. Induration of the Os and Cervix.*

Induration of the mouth and neck of the womb is not unfrequently met with, as an obstacle to the progress of labour. It appears to be the result of chronic inflammation, which has continued for some time unattended to, or uncontrolled by the remedies which have been employed. It may affect both limbi of the os uteri at once; or, what is more common, one only may be hypertrophied. The anterior is more frequently enlarged and indurated than the posterior: this, no doubt, is caused by its position rendering it more exposed to those causes which induce inflammation.

Mr. Lever points out the symptoms, which must be pretty familiar, of chronic inflammation of the os and cervix uteri. The following is the method of treatment that he recommends.

"This induration of the os uteri is met with in parturient women; and is occasionally a cause of obstructed labour, preventing its dilatation sufficient to permit the passage of the child. The treatment that I have employed, in such cases, consisted in venesection, the administration of tartar emetic, and the employment of the warm bath. Bleeding may be advantageously resorted to, where the patient is plethoric, and when she is not worn out by the continuance of the dilating pains of labour; but if she be of delicate habit, or if the labour has been of long duration, venesection will be found to predispose to subsequent uterine hæmorrhage: this remark accords with the experience of the most practical accoucheurs; amongst others, Dr. Ramsbotham, sen. Tartar emetic I regard as a most valuable remedy in these cases: it is a medicine which exercises a peculiar influence on all rigidities of the os uteri; and its effect is so completely to master all resistance, that it permits the necessary dilatation to take place: it is also a medicine so completely under controul, that if we find its nauseating effects too great or too long continued, we have only to tickle the fauces of the patient, and we at once cause her to reject the medicine, and excite re-action of the system by the production of vomiting. When administered in cases of induration, it should be given in doses sufficient to produce nausea: this nauseating effect must be kept up for some time; the period to be regulated by the altering condition of the os uteri, and by the general condition of the patient. When the dilatable state of the mouth of the womb has been produced, a full opiate should be administered, to maintain its relaxation, and to prevent that spasmodic and irritable condition of the os uteri which so frequently co-exists with induration. A recurrence of uterine action will then generally be successful, in completing the delivery.

The warm bath I have employed in but one case; but so pleased was I with its effects, that whenever I have the opportunity, I shall again order its employment in the obstruction under consideration. The great difficulty, however, is its application when ordered; as in but few houses do the means exist of heating water in so large a quantity, and so quickly as necessary."

Mr. Lever relates three cases, but it does not seem necessary to introduce them. Suffice it to say that nauseating doses of the tartar emetic, succeeded by an opiate, answered perfectly in two, while the warm bath had a surprising effect in the third.

*b. Abscess.*—Mr. Lever has seen one case of abscess of the portio vaginalis of the uterus, which occurred in a patient six months advanced in utero-gestation; and although the abscess evacuated its contents previously to the occurrence of preternatural labour, still the adhesive matter which had been thrown out, and which formed the walls of the abscess, offered a serious obstacle to the dilatation of the mouth and neck of the womb. Mr. L. relates the case in question.

*Case.*—Mr. Lever was requested to see Mrs. C. a lady 24 years of age, who was in the sixth month of her second pregnancy, having previously aborted at the fourth month. For a week she had suffered from constant acute pain in the passage and above the pubes, increased upon her assuming the erect posture, moving her thighs, and also during the expulsion of the contents of the rectum and bladder: the pain did not extend to the back or thighs, and there was no vaginal discharge. The pulse was quick and sharp; the skin hot and dry; the tongue furred; and the bowels, which had been opened in the morning, had caused her great increase of suffering. The fæces were very hard.

Twelve leeches were applied around the vulva, and above the symphysis pubis. The decoct. papav. was ordered to be frequently injected into the vagina; and castor oil, with saline medicine, was prescribed. Next day, twelve more leeches were applied.

"I was called to her early the next morning; and was informed, that, after passing a very bad night from excessive throbbing and pain, there had been a sudden copious discharge of pus mixed with blood: its quantity could not have amounted to less than three ounces, and its evacuation was followed by immediate relief. Internal examination was now for the first time permitted: the vagina itself was ascertained to be natural to the touch; but on the right side of the mouth of the womb a large cavity was found: this was the seat of an abscess, which had ulcerated, and burst into the vagina: the opening was so large, that it could not have altogether been caused by the process of ulceration."

The abscess discharged satisfactorily, and healed in about three weeks. This lady was now subjected to exertion and jolting in a carriage which induced great pain in the loins, back, and thighs, accompanied with discharge of blood from the os uteri. Notwithstanding all the means recommended and usually found of service in preventing premature labour were had recourse to, they proved of no avail; for in three days after the first appearance of these untoward symptoms, discharge of the liquor amnii took place, the uterine contractions returned, were regular, and of an excruciating character. The feet of the fœtus presented: after some considerable time, owing to the non-dilatability of the os uteri, the nates passed, but the greatest difficulty was experienced in the passage of the head. But little assistance could be rendered by traction, as the child was in so decomposed a state, that very little force would have been required to have separated the trunk and left the head in the uterine cavity. At length, during a powerful fit of vomiting, the head suddenly slipped from the uterus, followed by the placenta. Her convalescence was rapid. This lady has again been confined, and but little impediment was offered to the dilatation of the os uteri. Twelve months elapsed between the two labours.

Mr. Lever refers to another case related by Bonetus. The woman was the wife of a soldier, who was in labour for five or six days; and at last died, worn out with pain. The uterus being inspected, a large abscess, filled with very putrid pus, was found in the neck.

*c. Prolongation of the Anterior Lip of the Os Uteri.*

This state of parts, says Mr. Lever, generally occurs where there is a non-yielding of a portion of the os uteri; and the part more commonly affected is the anterior lip. In these cases, there is generally a small pelvis; and the head passes into the brim, before the os uteri is thoroughly dilated. The anterior portion of the os will then be forced down between the head of the fœtus and the pubes; and is prevented from being freed by the occurrence of successive pains, each uterine effort serving only to render the grasp more tight. The effect is soon perceived by the strangulated portion becoming œdematous; and if within view, it will be found of a dark colour. The uterine efforts are regular, and most commonly violently expulsive, short in duration, and terminate suddenly with a cry of despondency, which is well marked in the patient's looks and expressions. Mr. Lever cites a remarkable case reported by M. Duclos. He then proceeds:—"The shape of these enlargements is generally round: their length varies: it may not extend to more than an inch, or, as in the cases of Duclos, and one presently to be mentioned, it may project through the vulva. Their formation appears to depend upon the pressure to which the anterior lip is exposed, in a small pelvis, by the fœtal head: the circulation in the part becomes retarded; the veins cannot return the blood sent into them; effusion into the cellular tissue takes place, and consequent swelling of course, increasing in proportion to the duration of the pressure, and itself, in turn, becoming an obstacle to the onward progress of the child's head. The diagnosis of these swellings, if large, is at first not easy, especially if we have not had charge of the patient from the commencement, or can get but a brief and imperfect account of the os uteri at the commencement of labour. It may be distinguished from polypus, with which it is most likely to be confounded, by there having previously been no symptom of that disease; by its feel, form, and attachment. From the placenta it may be distinguished by the history of the labour, the os uteri being natural at its commencement; by there being no hæmorrhage, or any other symptom to mark the præviable placenta.

It is by no means uncommon to meet with those cases of prolonged anterior lip in a minor degree. The labours in which this condition is manifested are generally tedious, and there exists more or less non-yielding of the os uteri from the commencement. If, with the view of relaxing the os uteri, the ant. pot. tart. be exhibited, this prolongation does not usually occur; but where it is formed, when each successive pain serves but to increase its size, and where the patient's looks and expressions are assuming a character of despondency, the best plan of treatment that I am acquainted with, and one which I almost universally find successful, is the keeping two or three fingers fixed against the swollen anterior lip during the pains: these prevent further elongation; and I have generally found, that, after judicious pressure has been maintained for some time, the strangulated portion will slip up, and the head descend. By some writers we are recommended to puncture the swollen os uteri. I have performed this operation but once; where the elongated lip protruded through the os externum was of a dark colour, and very œdematous. In such cases, after delivery, fomentations should be persevered in for some time: the one I employ is the decoction of chamomile-flowers, to which a small portion of rectified spirit is added. The only inconvenience I have found to follow the prolongation of the anterior lip is the retention of the urine for two or three days, rendering the introduction of the catheter necessary. The state of the bladder must be attended to during labour, whenever this elongation exists to any extent."

Mr. Lever relates two cases in illustration.

*d. Hard or Fibrous Tumors of the Uterus.*

We see nothing particular in Mr. Lever's description of these.

Of the medical treatment of Fibrous Tumors we need say nothing. The following are Mr. Lever's sentiments on the surgical treatment. Some writers, says he, have recommended the removal of these tumors, by the operation of excision. Lisfranc has removed them, by making incisions through the cervix. If the tumor be limited to the portio vaginalis of the uterus, the operation of removal may be resorted to. An interesting case of this kind is related by Dr. Ingleby, which was removed by Mr. Evans, of Belper; but, as a general rule, it may be affirmed, that while these tumors are within the cavity, they will not admit of removal; but if they become pediculated, and pass through the os uteri, they may be treated in the same way as polypi uteri.

The operation of puncturing these tumors is of little avail, as, for the most part, they are solid: if they contain fluid, it is generally of a glairy, gelatinous, or grumous consistence; and the evacuation does not give rise to sufficient collapse to allow of the transmission of the child, when an obstacle sufficient to impede its birth is occasioned by the tumor. The operation of turning is useless, when fibrous tumors occasion such an obstacle to the birth of the child as to render artificial assistance necessary; not to mention the difficulty experienced in introducing the hand into the uterus, to grasp the feet. If called to a patient in labour, in whom such an obstacle to the passage of the child exists, we must make a careful examination, to ascertain whether we can safely leave the case to the natural efforts; but if we are convinced that the unaided powers of the womb will not be sufficient to expel the child, we must then determine whether the embryospastic or embryotomic instruments must be employed to complete the delivery. In such complications, we must not allow the labour to proceed too long before such assistance is given; as not only may the tumor be so bruised and compressed, that inflammation of its structure, followed by suppuration or gangrene, may take place, but also the uterus itself may give way; as in the case recorded by Fabricius Hildanus, c. 1, obs. 67. Sometimes the tumor is so large, that embryulcia cannot be performed; or, if the head is perforated, the child cannot be extracted through the small space. A case of this kind is related by Dr. Montgomery, at p. 188 of his valuable work on the Signs and Symptoms of Pregnancy.

Section of the symphysis pubis, recommended by some French writers, has not been practised in this country; and if performed, is not likely to be of any avail.

Induction of Premature Labour.—If a patient affected with a fibrous tumor become pregnant, and if that tumor be so situated, or be of such a size, that it will impede the progress of labour, it becomes a matter of great moment to determine whether or not the operation for the induction of premature labour should be performed. This operation, recommended by Akakia, in his Treatise on Female Diseases, has received the sanction of Dr. Ashwell.

Mr. Lever relates four cases which present no particular features.

*e. Polypus or Polypoid Tumors.*

Under this division, Mr. Lever includes all pediculated tumors not malignant, whether of a cellular, glandular, or fibrous texture, which, by their attachment to the uterus, impede or prevent the progress of labour.

We see nothing in his observations to detain us, unless it be one on the Ligature. "Obstetric writers and practitioners recommend ligatures of various kinds, as waxed silk, catgut, silver wire, silk wrapped round with wire, &c.; but no ligature, in my opinion, is preferable to whipcord: it has been shewn by Mr. Walne, in the Medical Gazette, July 1836, that whipcord, when moistened, increases in thickness, and diminishes in length: thus, such a ligature, after its application, and when bathed in the discharge, will tighten itself very considerably. The canula I employ is the one in use at Guy's Hospital: it consists of Gooch's canula, to the outer end of which a rack has been superadded, so that the ligature may be gradually tightened from time to time by turning the rack, instead of unwrapping the end of the ligature and drawing it tighter, as was necessary in the original instrument."

Mr. Lever proceeds to

MALIGNANT DISEASES ASSOCIATED WITH PREGNANCY.

*Carcinoma Uteri.*

Mr. Lever remarks that, so far as he has seen, the encephaloid form of carcinoma is more frequently found in younger females than the scirrhous variety.

The part of the womb most frequently attacked by scirrhous disease is the neck. Some writers, as Sir C. M. Clarke, Blundell, &c. attribute this to the cervix being the most glandular part: others, as Wenzel, admit the fact, but explain it by stating the cervix is more exposed to injury. Puchelt has collected thirty-two cases of labour complicated by scirrhus uteri, where the seat of disease was as follows:

| | | |
|---|---|---|
| The whole uterus was scirrhous in | 1 | case. |
| A large portion of the organ.. .. | 5 | cases. |
| The neck of the uterus .. .. | 11 | .... |
| The neck and mouth .. .. .. | 5 | .... |
| The mouth alone .. .. .. .. | 6 | .... |
| The left side .. .. . .. .. | 1 | .... |
| The body .. .. .. .. .. .. | 1 | .... |
| The fundus .. .. .. .. .. .. | 2 | .... |
| Total .. .. | 32 | cases. |

Passing the account of the appearances which carcinoma presents, we may advert to Mr. Lever's notice of its prognosis in connection with pregnancy:—1st, the prognosis with respect to the mother, and, 2dly, the prognosis with regard to the child. With respect to the mother, much depends on the method of delivery. If the scirrhous formation be in its early stage, if it be small in size, and if the patient's constitutional powers have not been too much lowered, the woman will generally go through her labour; although such process may be rendered lingering, by the pressure of such a tumor: and even when the child has been delivered with instruments, the patient will, for the most part, recover the immediate shock of parturition, to die at an earlier or later period, from the effects of the malignant disease. But it must be allowed, that all labours, whether terminated naturally or artificially, cause the progress of the malignant disease to be more rapid. Of twenty-seven women, Puchelt informs us five died during labour, nine a short time after labour, ten recovered, and in three cases the results were not known. Abortion not unfrequently takes place when there is malignant disease of the uterus. The prognosis with respect to the life of the child is generally unfavourable; for the length of the labour, superadded to the compression which the child's head has to undergo when the carcinoma exists at the mouth or neck of the womb, where the patient is delivered by the unaided natural efforts or by the use of the embryospastic instruments, is generally sufficient to cause the death of the child. In the twenty-seven cases previously referred to, fifteen children were still-born, ten were born alive, and in two nothing is stated respecting the viability of the child.

For the benefit of such of our readers as are likely to be placed in such embarrassing circumstances, we are tempted to extract Mr. Lever's observations on the treatment. "In determining," says he, "the method of treatment to be adopted in a case of pregnancy combined with malignant disease of the mouth or neck of the womb, we must carefully examine the seat, form, and size of the opposing obstacle; ascertain, if possible, and with as much accuracy as the case will admit, the dimensions of the pelvis; and then cautiously determine in our minds the chances of delivery occurring at the full period, by the unaided efforts of the uterus. In a case to which I shall presently allude, and where the tumor occupied the posterior part of the cervix, the labour, although rendered lingering, was terminated by the unaided uterine efforts.

Such instances, however, are rare; and artificial assistance of various kinds is necessary. Some authors recommend emollient injections:—these are of no avail. Others extol large blood-lettings:—venesection, although of the greatest advantage in simple rigidity of the os uteri, and induration of the os and cervix, the result of chronic inflammation, will be found of little or no value in carcinomatous affections of the uterus. Others, as Madame Boivin and Dr. Ashwell, recommend incisions to be made in the diseased parts, to permit the passage of the child: and it cannot be denied, that had such an operation been performed in many of the recorded cases where the patients have died undelivered, either from rupture of the uterus or collapse, the chances are that the mothers' lives would have been prolonged, and the viability of the children secured. Some authors, as Lieutaud, Dugès, &c. recommend the extirpation of the tumor by the knife or cautery: this practice, however, it not adopted in this country; neither, in my opinion, is it at all practicable, much less adviseable. Siebold recommends that version should be employed in such complications as these under consideration; but to introduce the hand into a uterus so much affected with malignant disease as that it is not considered prudent to leave the case to the natural efforts, and then to grasp and bring down one or both feet, would necessarily be attended with much violence and laceration, and the passage of the fœtal head would still present a great difficulty. It is, moreover, very unlikely that such an operation will become successful, as it regards the life of the child; for, in addition to the causes operating to render its viability doubtful, which is the case whenever version is performed, we have the additional one of the retardation produced by the non-dilatability of the soft parts through which it has to pass. Cases are recorded where version has been performed, and, in order to expedite the delivery, more force than usual has been employed; but the patients have died almost immediately after delivery, from either rupture of the uterus or from collapse. The forceps will frequently be of great assistance, especially in those cases wherein there is but one, or it may be two scirrhous tubercles, and these not in an advanced state. A case presently to be related, and for the particulars of which I am indebted to Mr. Butler of Woolwich, is a very fine example of their application; and even here, Mr. B. delayed their employment until the natural efforts were allowed to do their utmost to accomplish the delivery. In cases where there is such structural change, that the application of the forceps is impracticable, and where the child is indisputably alive, as proved by its movements and by the auscultatory signs, it is of great importance to determine whether we are justified in opening that child's head and destroying its life, or whether we should perform the Cæsarean section. This is a question which I think demands consideration. In many cases on record, I am of opinion that the life of the child might have been spared, if such an operation had been had recourse to; whilst several of the mothers died during labour or soon after delivery; and in others, their miserable existence was prolonged but for a few weeks."

### TUMORS IN THE VAGINA.

Mr. Lever treats of these under the following heads.

### *Abscess of the Vagina.*

Mr. Lever has seen two cases of abscess in the vagina during pregnancy: both the patients were prostitutes, and both were prematurely confined: they had laboured under gonorrhœa, and had used stimulating injections: one had injected a strong solution of bichloride of mercury; the other had used the lotio nigra. The abscesses were opened during the time they were in labour; and in each case a considerable quantity of most fetid pus was evacuated. Probably, if an artificial opening had not been made, the uterine efforts would have sufficed to cause laceration of the vaginal wall of the abscess; which in one case was thinning in the centre; in the other case, nearer to the outlet.

### *Polypus of the Vagina.*

Mr. Lever believes that polypoïd tumors more frequently grow from the anterior than from the posterior wall of the vagina or its sides. The diagnosis of the tumors is not difficult, unless they have acquired a size sufficient to prevent the introduction of the finger to detect the part from which they spring. The treatment of these tumors, when discovered during pregnancy, should be as follows:—If they are of small size and are not likely to impede the passage of the child, and if they do not produce any untoward or urgent symptoms, they may be left until the patient is recovered from the effects of parturition; but if their size is of such dimensions, that great inconvenience and protraction of the labour is likely to result, or if there is hæmorrhage or any other unnatural symptom, their removal must be undertaken. Unlike polypus uteri, polypoïd tumors of the vagina seldom, or never, become less, after labour has taken place. The former progresses with the development of the pregnant womb; while the latter, being attached to and growing from, the vagina, has not that greatly-increased vascular supply which polypus uteri has in common with the impregnated organ.

Mr. Lever thinks that the best operation is the combination of the ligature and excision. This he has performed two or three times on patients who have not been pregnant; and once, on a patient six months advanced in utero-gestation.

### ENCYSTED TUMORS OF THE VAGINA.

These occasionally interfere with labour. They are not confined to either wall of the vagina; the anterior is as obnoxious to them as the posterior; when they form on the latter, they are likely to be mistaken for hernia, enlarged ovary, &c.; but a careful examination will readily enable us to determine their nature. When they form on the anterior wall, they have been mistaken for descent of the bladder: here also cautious examination, and the introduction of the catheter, will enable us to decide upon their seat and origin. If these tumors are discovered before labour, they may readily be dissected out entire, through the vagina: this operation, although attended with considerable hæmorrhage, is seldom dangerous; and the bleeding is controllable, by plugging the vagina. If the tumor be not discovered until the process of labour is established, an incision into it, through the vagina, will allow its contents to escape, and remove the obstruction.

### MALIGNANT DISEASE OF THE VAGINA.

Where malignant disease is associated with pregnancy, it is a question of great moment to decide whether we ought to induce premature labour, in order that the body which has to pass the narrowed birth-passages should be proportionably small, or whether we should allow the patient to go on to the full period of utero-gestation. If the latter be decided on, we shall have to determine whether we are justified in destroying the child by opening its head, or whether the Cæsarean section should be performed. Mr. Lever is inclined to prefer the latter. If it be determined to sacrifice the life of the child, whatever be the stage of the disease, it is far preferable to induce premature labour than wait till the completion of pregnancy. If the disease be not detected until the occurrence of labour, the surgeon must determine whether the case may be trusted to the natural efforts, or whether artificial assistance is to be given.

### TUMORS DEPENDING UPON ACCIDENTAL CAUSES.

#### *Sanguineous Tumors or Thrombi.*

Thrombi are sanguineous tumors, which form in the labia or vagina, covered by mucous membrane, and produced by the rupture of a vein, and consequent effusion of blood into the cellular tissue. These tumors sometimes attain such a size, that, by their magnitude, they impede labour. "The extravasation of the blood into the cellular tissue usually occurs at the time when the child's head is expelled; but in the only case I have seen (the one presently to be related) it occurred while the child's head was yet in the pelvic cavity, and before it pressed upon the perinæum. In such cases there is generally a small and contracted pelvis, or the child's head may be large or firmly ossified: it arises from the return of blood being prevented by the pressure of the presenting part of the child; and the pains continuing violent, the distended veins become more and more swollen, and at length the distention being increased, the vessels give way, the blood is poured into the cellular tissue, the quantity effused becomes greater and greater, the tumor proportionately increases, till at length the thrombus becomes of such a size as to impede the progress of the labour. The sudden formation of the tumor, its rapid increase, its gradual enlargement at every successive pain, and the sensation communicated to the finger, will readily enable us to distinguish between this tumor and abscess, encysted tumors, &c. If the thrombus is formed while the child's head is in the pelvic cavity, the pressure made upon the tumor during the birth of the child generally causes the mucous membrane to give way: the contents of the tumor are then evacuated: they consist of fluid and coagulated blood, and the quantity which is sometimes let out is very considerable. In Dr. Ingleby's case, the coagula filled a pint basin; while a considerable quantity was still left, and occasioned sloughing. In a case related by Zeller, the tumor was of the size of a fœtal head. I have stated, that, in many cases, these tumors form at the time the child's head is expelled, and of course cannot then offer any impediment to the labour: when, however, they form while the head is still engaged in the pelvis, where the cavity is very small, and when the child's head is large or extraordinarily ossified, more or less obstruction will be caused by the formation of such a tumor. If the mucous membrane covering the tumor give way, the greater part of the contents will be evacuated; and if the effusion be large, it will lessen the strength of the patient, especially if her powers have already been enfeebled by her lingering labour. The results of the published cases enable us to conclude that the effects of these swellings are not generally fatal, either to the mother or the child. Where a thrombus exists of a size sufficient to obstruct the progress of the child's head, an incision should be made sufficiently large to evacuate the contents of the tumor: if this is not done, the mucous membrane covering the tumor will give way, the cellular tissue will become torn and inflamed, and this inflammation will frequently terminate in sloughing.

To accomplish delivery, version was employed in one case related by Deneux, p. 23; the forceps in a case related by Siebold; the vectis was used by Zeller; and the perforator" in a case related by Mr. Lever himself.

Mr. Lever also relates an interesting case of erectile tumor of the labium. He goes on to say:—" The vagina is sometimes covered with large dilated varicose veins: these rarely offer any obstruction to the progress of labour. One of the evils I have known result from a varicose state of the vaginal veins, is, that coagulation of the blood sometimes takes place, followed by inflammation and abscess, not sufficient to arrest the progress of the labour, but increasing to a very great degree the sufferings of the patient. One case of this kind I have witnessed, where the agony endured by the patient was almost insupportable. Another result of varicose vaginal veins is, laceration of one of the enlarged vessels, and consequent copious hæmorrhage. To a case of this kind I was once called: the patient was delivered before my arrival, and had lost a considerable quantity of blood: bleeding was still going on, but not to a great degree. Her labour had been protracted, the mother having a small but regularly-shaped pelvis; and the child, a large, firmly-ossified head. Pressure with a dossil of lint was made over the laceration, which was not very large, and the bleeding was stayed. The patient was anæmiated for a very long period."

Mr. Lever details a case in which labour was much obstructed by *warts*. The suffering was excessive. After the labour, the soft parts became hot, swollen, and inflamed; and on the fourth day the warts commenced sloughing off, and by the seventh day were entirely separated.

### IV. Two Cases of Injury to the Head, followed by Symptoms of Compression produced respectively by Extravasation of Blood and Formation of Pus relieved by Operation. By Edward Cock.

Speaking of compression, Mr. Cock remarks:—It is almost invariably preceded or accompanied by concussion, and may depend on various causes—on depressed bone, on extravasation of blood, or, at a subsequent period, on the formation of matter within the cavity of the cranium; or again, on a combination of these causes. When bone is depressed, the appearances of external violence will generally indicate the seat of the injury, and suggest the propriety of making a further investigation; but this is by no means necessarily the case where compression ensues from extravasation of blood or from suppuration. A host of difficulties then surround the case, and embarrass the diagnosis. Blood may be poured out between the dura mater and the skull, or within the cavity of the arachnoïd; or it may be effused over the surface, or deeply within the substance of the brain itself. It is the first of these only that we can expect to relieve by an operation; and our success will then probably depend on the absence of any other important lesion to the contents of the cranium. The surgeon may be called on to determine, 1st, whether the compression is produced by extravasated blood; 2dly, in which of the three situations, as regards the brain and its membranes, the blood has been poured out; and 3dly, the spot where the trephine should be applied, if an operation be deemed advisable. It not unfrequently occurs, that symptoms of compression become manifested, without any well-marked local indication of the seat of mischief; that the most careful examination fails in detecting the appearances of external violence; that the history of the accident is defective in affording us the desired information; that the surgeon is thus thrown entirely on the resources of his physiological and pathological information, and must decide, from the progress of the symptoms and the effects produced on the different parts of the patient's frame, whether the mischief is of such a nature, and so located, as to admit of relief by application of the trephine.

Mr. Cock relates two cases. They are both interesting. One was that of a man who, after a fall, had stertorous breathing, &c., and paralysis of the right side, with severe scalp wound over the left parietal bone. Mr. Cock cut down but could find no fracture. He then proceeded to remove a portion of the bone above and behind the anterior inferior angle with a full-sized trephine. The moment the elevator had detached the bone, a gush of blood took place through the opening, and a clot was discovered beneath. On passing the finger into the cranium, it entered an extensive mass of coagulated blood, which extended, in every direction, as far as the finger could reach, and was evidently of considerable depth. The interior of the cranium, as far as could be felt, presented no irregularity or trace of fracture. A small quantity of the coagulum was removed with the handle of a tea-spoon, and the wound was then covered with lint dipped in water. The deep stertor of his breathing had ceased almost on the instant that the bone was raised, and he was evidently relieved by the operation. Suppuration ensued between the bone and dura mater, but the patient ultimately recovered most satisfactorily.

Mr. Cock thinks he has noticed that in every case of extravasation from rupture of the middle meningeal artery, the pure symptoms of compression are more distinctly and strongly marked than where they are occasioned by depressed bone, or effusion of blood in any other situation:—there is a death-like quietude of the limbs; the face is devoid of expression or muscular motion; the powers of the patient seem concentrated to produce the laborious heavings of the chest; while the lips and cheeks are mechanically puffed out, as the air is expelled from the mouth at each expiration. These signs, together with the deep loud tone of the stertor, are, I think, very characteristic. The pupil, also, on the same side as the extravasation, will generally be found dilated; although this was not the case in the present instance. These peculiarities may probably be attributed to the clot of blood being to a certain degree circumscribed in extent; to its great depth; and the pressure which it consequently exerts on the opposite surface of the brain, which becomes deeply impressed by it: whereas, when blood is effused into the arachnoid cavity, it is generally less in quantity and spreads over a much wider surface, producing more general and less local pressure. When extravasation takes place on the surface or within the substance of the brain, it is accompanied and indeed produced by lesion of the cerebral texture; which lesion is mostly indicated by paralysis, by irritation or spasmodic action affecting some particular part, by derangement of the pupils, or by other symptoms.

Mr. Cock adds :—A case very similar to that which forms the subject of this Paper occurred a few years ago in the hospital; and its result gave me great reason to regret that I had not trephined the patient. On making a visit to one of the wards late in the evening, my attention was attracted by the deep stertorous breathing of one of the patients; and, on inquiry, I was informed it was a man who was then under treatment for apoplexy, having been admitted, in a comatose state, that afternoon. The history was briefly this. He had been brought to the surgery in the morning, in a state of syncope, having, as was reported, fainted in the street. Some stimuli were given him, under which he speedily rallied and walked away. In the course of the afternoon he was again carried into the hospital, in a state of complete coma with apoplectic stertor. The usual treatment—bleeding, calomel, &c.—had been practised without relieving his symptoms, and he was evidently in a hopeless state. The details of the case and the general appearance of the patient, induced me to suspect that he might be labouring under extravasation of blood beneath the cranium, the consequence of injury produced by a fall, the result of accident; and not, as had been supposed, the result of cerebral effusion. On examining the head, I discovered a slight bruise on the right temple; and determined to cut down on the bone in that direction, on the speculation of finding a fracture. However, after exposing the angle of the parietal bone, no trace of fracture was discoverable, and I did not feel justified in proceeding further. He died in the course of a few hours; and, on examination, a minute fracture, hardly discernible on the exterior of the cranium, was found to traverse the lower extremity of the parietal angle. A large clot of blood had accumulated between the dura mater and the scull, poured out from the ruptured meningeal artery. There was no other injury whatever to the contents of the cranium.

Mr. Cock relates another case in which, after a kick from a horse, the frontal bone was fractured immediately above the nasal process a portion of brain escaped. There were no unpleasant symptoms for a fortnight, when the lad became comatose, and the wound was dry and unhealthy. This was succeeded by fever, irritability, screaming. On the 20th day, he became hemiplegic on the left side. The loss of motion and sensation was complete in the upper extremities. The paralysis of the leg was less decided in character: the mouth was drawn to the right side. There was occasional convulsive twitching of the muscles of the arm and face. At times, he appeared to be labouring under all the symptoms of compression, but at intervals was perfectly sensible. He had been salivated. He was leeched.

Next morning, the convulsive attacks, which had manifested themselves the day before, were renewed with increased violence. The muscular spasm and twitchings of the left arm and left side of the face became incessant and intense: his pulse quick and feeble, but irregular. These symptoms continued, without intermission, for some hours; and in the afternoon, the chances of his recovery seemed hopeless, unless some relief could be obtained.

"As the progress and nature of his symptoms were such as indicated the formation of matter either within the substance of the brain or between the membranes, I determined to make an attempt to discover the seat of mischief, by removing a portion of the frontal bone. With the Hey's saw, I cut through the bone, in two convergent lines carried upwards from the track of the fracture for about the length of an inch, where they met. A triangular piece of the os frôntis was thus removed, just on the right side of the median line. The dura mater, which was now exposed, bulged slightly forwards, and was devoid of pulsation: I divided it freely, by a crucial incision. A small quantity of semi-opaque, dirty-looking serous fluid appeared to escape from under the membrane; but its character and quantity could not be very accurately ascertained, as it became mixed with blood from the dura-matral vessels. The surface of the brain, now brought into view, was disorganized and broken up, apparently in that stage of softening and disintegration which precedes the formation of an abscess. I passed a director for a short distance through the pulpy tissue, in hopes of discovering a collection of pus; but failing in this, considered it best to abandon the search, and remain satisfied with having removed any impediment that the bone might have offered to the future escape of the fluid, trusting that Nature would now accomplish the rest. The wound was covered with warm-water dressing. He complained loudly during the operation, which seemed to have the effect of rousing him from his stupor. Shortly after, he again became convulsed; and his left arm, the left side of his face, and chest, continued to be violently agitated for about two hours: he then became tranquil; and had had no relapse, when I again saw him, at 11 o'clock the same evening.

By the 24th day healthy-looking pus was discharged from the opening in the brain. By the 26th day, he could move the left arm and leg freely. Feverishness now came on again. On the 28th day there appeared swelling of the left upper eyelid. It was evident that the eyelid was protruded by a collection of fluid within the orbit, and situated behind the tarsal cartilage, as there was no infiltration into the cellular texture of the lid itself. A free incision was made through the tarsus, which allowed the escape of a quantity of matter similar in character to that which had been evacuated from the opening in the brain. The symptoms of irritation speedily subsided; and the next day, on introducing a probe through the opening, I could distinctly feel a fissure extending across the roof of the orbit, through which the matter had probably found its way from the cavity of the cranium. A fungus cerebri, which formed, was repressed by gentle pressure aided by an occasional touch of caustic, and the patient got quite well."

Both these cases are creditable to Mr. Cock.

### V. OBSERVATIONS ON URINARY CONCRETIONS AND DEPOSITS; WITH AN ACCOUNT OF THE CALCULI IN THE MUSEUM OF GUY'S HOSPITAL. By GOLDING BIRD, A.M., M.D., &c.

Of Dr. Bird, it is not necessary for *us* to speak. We have ever been happy to express our high opinion of his talents and acquirements. He has but to work steadily and quietly, and his place as a chemical physician is sure to be a very high one.

It is now a quarter of a century since Dr. Marcet published an account of the urinary calculi, in the museum of Guy's Hospital. It then contained 228. During the last twenty-five years, this number has been augmented to 363; all of which have been divided so as to exhibit their internal structure, with the exception of 21. The great majority of the calculi added since Dr. Marcet's publication have been analysed at different periods, as they were placed in the Museum, by Dr. Babington, Dr. Rees, and Dr. Bird. In every instance the examination has not been limited to the composition of the external crust, but has been particularly directed to the chemical constituents of the ingredients composing each layer. Attention has in every instance been particularly directed to the composition of the nucleus, in contradistinction to that of the body of the concretion. This is of very great importance; for when once a few solid particles of any substance aggregate and form a mass in the bladder, they very readily induce a crystallization of oxalate of lime, uric acid, or triple phosphate; or a deposition of urate of ammonia, phosphate of lime, or other amorphous ingredient, according to the lesion of function and state of irritability or innervation present. Hence, if ever, by medical treatment, we shall be enabled to prevent the formation of a calculous concretion, or remove one already formed, it will, in all probability, be by means directed by the character of the matter which there is a tendency to deposit as a nucleus. Dr. Bird has, on this account, adopted a classification of the calculi in Guy's Hospital Museum, founded not upon the number of alternating layers, but upon the character and composition of the nucleus. In the following Table, it must be borne in mind, that all the distinct constituents present in each concretion have not been mentioned; those only being inserted which were present in such quantity as to constitute a considerable portion of either body, nucleus, or crust of the concretion. Those ingredients, which existed in mere traces, or in very minute quantities, have been omitted; as they are rather to be regarded as accidental contaminations, and not as essential elements of the calculus. No urinary concretion, indeed, ever exists perfectly pure and unmixed; for there are very few in which some traces of uric acid, or phosphates, are not observable: and even if these be absent, the colouring matter of urine or blood prevents the calculus being regarded as perfectly pure.

Dr. Bird subjoins a Table which appears to us a very valuable and useful one.

CALCULI IN GUY'S HOSPITAL MUSEUM,

Of which sections have been made, arranged accordiug to the chemical composition of the nuclei.

GENUS I.—NUCLEUS, URIC ACID, 245.

Species I. *Calculi nearly entirely composed of Uric Acid or Urates.*

| | | |
|---|---|---|
| *A.* Nearly all uric acid | | 31 |
| Uric acid, nearly pure | 18 | |
| Stained with purpurine | 2 | |
| Contained urate of lime | 2 | |
| and ammonia | 2 | |
| urate of soda and lime | 1 | |
| oxalate of lime | 3 | |
| phosphate of lime | 1 | |
| triple phosphate | 2 | |
| | 31 | |
| *B.* Body consisting chiefly of urates | | 169 |
| Contained traces of urate of soda | 142 | |
| and lime | 22 | |
| urate of lime | 4 | |
| uric acid in the body | 1 | |
| | 169 | |

Species 2. *Bodies differing in composition from Nuclei.*

| | | |
|---|---|---|
| *A.* Bodies consisting of oxalate of lime | | 10 |
| Oxalate of lime and uric acid alternating | 2 | |
| Uric acid in the body, with an outer layer of carbonate of lime | 1 | |
| Oxalate, chiefly confined to external layers | 1 | |
| Oxalate of lime in the bodies nearly pure | 6 | |
| | 10 | |
| *B.* Bodies consisting chiefly of earthy phosphates | | 22 |
| Bodies composed of fusible calculus | 14 | |
| phosphate of lime | 3 | |
| triple phosphate | 5 | |
| | 22 | |

| | | |
|---|---|---|
| *C.* Body consisting of carbonate of lime . . . . . | 1 | 1 |
| *D.* Alternating calculi . . . . . . . . . . . . . . . . . | | 12 |

| Body: | Crust: | |
|---|---|---|
| Urate of ammonia . | Fusible . . . . . . . | 1 |
| Oxalate of lime . . | Uric acid . . . . . . | 3 |
| | Fusible . . . . . . . | 3 |
| | Triple . . . . . . . | 1 |
| | Phosphate of lime . . | 3 |
| Fusible . . . . . | Uric acid . . . . . . | 1 |
| | | 12 |

GENUS II.—NUCLEUS, URATES OF AMMONIA OR LIME. 17

Species 1. *Calculi nearly all composed of Urate of Ammonia* . 7

| | |
|---|---|
| Urate of ammonia, nearly pure . . . . . | 5 |
| Uric acid, in tubercular patches on crust . . . | 1 |
| Traces of urate of soda and phosphate of lime . | 1 |
| | 7 |

Species 2. *Bodies differing from Nuclei.* . . . . . . . . . 9

| Body: | Crust: | |
|---|---|---|
| Uric acid and fusible. . | As body . . . . . . | 1 |
| Urate of ammonia . | Uric acid . . . . . . | 1 |
| | Oxalate of lime . . . | 1 |
| | Phosphate of lime . . . | 1 |
| and oxalate of lime . | Uric acid, with oxalate and phosphate of lime | 1 |
| Urate of ammonia and fusible . . | As body . . . . . . | 1 |
| Urate and phosphate of lime . . . . | Ditto . . . . . . . | 1 |
| Oxalate of lime . . | Fusible . . . . . . . | 1 |
| Fusible . . . . . | As body . . . . . . . | 1 |
| | | 9 |

Species 3. *Nucleus, Urate of Lime.*

| | | |
|---|---|---|
| *A.* Body fusible . . . . . . . . . . . . . . . . . | 1 . | 1 |

GENUS III.—NUCLEUS, URIC OXIDE.—*None.*

GENUS IV.—NUCLEUS, OXALATE OF LIME. 45.

Species 1. *Calculus, nearly all Oxalate* . . . . . . . . 18

| | |
|---|---|
| Uric acid in nucleus . . . . . . . . . . . | 1 |
| Crust, covered with opaque octohedral crystals . | 1 |
| transparent . . . . . . | 2 |
| not covered with crystals . . . . . . | 14 |
| | 18 |

Species 2. *Bodies differing from Nuclei.*

*A.* Bodies consisting of uric acid or urates . . . . . . . 8

| | | |
|---|---|---|
| Uric acid, nearly pure | 7 | |
| covered with urate of ammonia | 1 | |
| | 8 | |
| *B.* Bodies consisting of phosphates | | 13 |
| Phosphate of lime | 6 | |
| Triple phosphate | 4 | |
| Fusible mixture | 3 | |
| | 13 | |
| *C.* Body compound | | 6 |

| Body: | Crust: | | |
|---|---|---|---|
| Uric acid | Fusible | 2 | |
| | Oxalate of lime | 1 | |
| Urate of ammonia | Phosphate of lime | 1 | |
| 1. Uric acid<br>2. Oxalate of lime<br>3. Uric acid | Oxalate of lime | 1 | |
| Cystic oxide | | 1 | |
| | | 6 | |

GENUS V.—NUCLEUS, CYSTIC OXIDE.

| | | |
|---|---|---|
| Species 1. *All Cystic Oxide* | | 11 |
| Colour, greenish blue | 1 | |
| dirty greenish grey | 9 | |
| fawn brown | 1 | |
| | 11 | |

GENUS VI.—NUCLEUS, EARTHY PHOSPHATES. 21.

| | | |
|---|---|---|
| Species 1.—*All Phosphates of Lime* | 2 | 2 |
| Species 2.—*All Triple Phosphates* | 1 | 1 |
| Species 3.—*All Fusible Mixed Phosphates* | 18 | 18 |

GENUS VII.—INGREDIENTS OF CALCULUS MIXED WITH NO EVIDENCE OF ARRANGEMENT IN CONCENTRIC LAYERS . . . 3

| | | |
|---|---|---|
| *A.* Uric acid and triple | 1 | |
| *B.* phosphate of lime | 1 | |
| *C.* urates of soda and ammonia, with oxalate and phosphate of lime | 1 | |
| | 3 | |

ABSTRACT VIEW OF NUCLEI.

| | |
|---|---|
| Nuclei, consisting of uric acid or urates | 262 |
| cystic oxide | 11 |
| oxalate of lime | 45 |
| phosphates | 21 |
| | 339 |
| Mixed calculi | 3 |
| | 342 |

Dr. Bird has not included the fibrinous calculus of Dr. Marcet, as it must rather be looked on as a portion of dried inspissated albuminous matter exuded from an irritated kidney.

Among the other ingredients existing in calculi, in very minute quantities, and not enumerated in the Table, are, hydrochlorate of ammonia, oxide of iron, and carbonate of lime.

"Calculi present the greatest possible variety in appearance; generally, however, having more or less of an ovoid figure. Of those in Guy's Museum, the urate of ammonia and uric-acid concretions are the most regular, nearly all being ovoid or circular, a few only reniform; this species never presenting any very prominent processes or projections, unless fresh centres of deposition occur on their sufaces, as when crystals of uric acid are deposited on an ovoid urate of ammonia concretion. The cystic-oxide concretions vary considerably in outline; when large, being generally oval and smooth; and when smaller, often presenting projections from their surfaces, as if they were made up of crystals radiating from a common centre; sometimes being moulded to the figure of the organ which secreted it, as shewn in the curious ear-drop-like concretion. The oxalate of lime is generally most irregular, as far as the surface is concerned; although its outline is generally tolerably defined, either bearing a close approximation to an elliptic, or even a rectangular figure. The most contorted and irregularly-figured calculus is the triple or fusible, it being often a complete cast of the pelvis and calyces of the kidney; occasionally, however, it is almost regularly oval, and sometimes circular; this variation, in all probability, depending upon the position occupied by the calculus, and upon whether it had been retained in the kidney, or passed down the ureter before it had become of any considerable size. The mixed calculi, or those not presenting any regular concentric arrangement or a distinct nucleus, are often moulded to the kidney. The phosphate of lime calculus is generally smooth externally, and conchoïdal in fracture, sometimes appearing as if made up of several cohering portions. The triple phosphate and fusible mixture are not unfrequently found deposited on one side of a previously-formed calculus, as if one surface only had been exposed to the urine containing the earthy salt in solution, which is then generally found under the form of elegant white vegetations.

The nucleus is usually found in the geometric centre of the calculus, or nearly so; sometimes, however, being remarkably excentric, as in some reniform concretions; and in a few, several distinct nuclei or centres of deposition are met with. In some rare instances, the concretion which forms the nucleus is found loose within the body of the entire calculus; a circumstance in all probability arising from a layer of blood or mucus having concreted around the nucleus, and on which the matter forming the body of calculus became deposited. In this case, on the whole becoming dry, the mucus or blood would be diminished to a very thin layer, and the calculus would appear to contain loose matter in it. In a few instances, calculi appear to possess no nucleus, the centre being occupied by a cavity full of stalactitic or mammillated projections, giving the idea of the external layer having been first formed, and the mammillated portions subsequently formed in the interior. This state occurs only, so far as I have seen, in uric-acid calculi. In one specimen in the collection, the central cavity is lined with fine crystals of triple phosphate, resembling the crystals of quartz so often found lining cavities in flints. Brugnatelli describes one of a similar kind.

Sometimes calculi present very remarkable appearances, as if they had been divided into segments: this, in some cases, can be explained by the attrition of calculi against each other, where several exist at once. In some, they actually appear as if they had been divided by a fine-cutting instrument; and in one, in the Museum, the apparently divided portions seem as if they had again become cemented and framed in by a subsequent deposit: I confess myself quite at a loss to explain many of these very remarkable appearances."

Dr. Bird speaks highly of the assistance he has derived from the microscope. If it only aids, he observes, in the detection of a previously-overlooked deposit of oxalate of lime, it can scarcely be said to have been used in vain. A low power is sufficient for the examination of urine: a good half-inch achromatic object-glass, with a low eye-piece, is sufficient for the examination of every case of crystalline sediments.

Dr. Bird examines seriatim the different forms of calculous deposit, and offers an account of each which will repay the reader's careful perusal. He concludes the examination by some general remarks of much ingenuity and interest. He says:—" I would suggest the probability of the necessary existence of but two great classes of deposits, or diatheses in which they exist, instead of several different and distinct species of diseased action. I would venture to propose the division of deposits into two genera or classes; the first being organic, strictly the product of vital agency, not derived from without, and indebted for their origin to deranged function; the second, including those sediments which are wholly or in part derived from without, and often rather to be considered the result of wear and tear of the sane organs of the body than of active disease.

The first of these classes will include uric acid and urates, being the nearest approach to a healthy state of secretion. If, from any cause, less oxygen be eliminated, we have these replaced by the xanthic (uric) oxide; whilst, if the oxidating function of the kidney be exalted, oxalic acid is developed under the form of oxalate of lime. The tendency to an excessive elimination of sulphur will cause the replacement of uric acid, or urea, by cystine. To this category is also referrible the carbonate of lime, which I regard not as a secretion *de novo*, but a secondary product; the result of so great a state of enervation, that the bond of affinity, which in the healthy condition of the nervous system keeps firmly united the elements of urea, becomes loosened, a re-arrangement of atoms occurs, the urea becomes carbonate of ammonia, and, under the influence of ordinary chemical affinity, carbonate of lime is generated at the expense of the calcareous salts of the urine.

The second class contains the phosphates simple and mixed: the acid of these compounds may probably be really generated in the kidney by the oxidation of the phosphorus present in the fatty matter and albumen of the blood; and, if so, the bases are alone derived from without. Under certain circumstances, it is pretty evident that the earthy phosphates are developed by a sort of vicarious function of the kidney; for in some few cases, in which the phosphate of lime has not been deposited in the osseous structure, as in mollities ossium and rachitis, the urine has been loaded with it. *Cæteris paribus*, of the two phosphates, the salt of lime appears more generally the result of enervation, and the magnesian combination the product of irritation.

If these views be substantiated—and I trust soon to bring evidence before the Profession of their probability—they would tend to simplify the generally-received doctrine of the existence of a series of distinct diatheses—a doctrine, of the correctness of which I confess myself by no means satisfied. To take a single instance:—If, from the mal-performance of any function, as that of the skin by the suppression of perspiration, the kidney be called upon to perform a supplementary or compensating duty, for the purpose of excreting that amount of carbon and nitrogen for which the skin is temporarily unfitted, we know that the presence of urate of ammonia in the urine is the immediate result. It is surely, then, not making too great a call upon one's imaginative powers, to conceive that, under slight predisposing causes or idiosyncrasies, this deposit may become replaced by the other organic ingredients, or even by carbonate of lime, according to the comparative amount of irritation or enervation present in the system in general or kidney in particular. I need hardly allude to the influence these views, if proved to be correct, would have on the treatment of calculous affections. I, however, merely thus briefly allude to them; trusting to the forbearance of the critic, until I have an opportunity of making known the data on which I have ventured to advance them." We confess that we look forward with some interest to the development of these views of Dr. Bird.

### VI.—ON THE LOCATION OF PULMONARY PHTHISIS, AND ITS RELATION TO DIAGNOSIS. BY H. MARSHALL HUGHES, M.D.

Dr. Hughes has, with great pains and precaution, constructed a Table, with a view to shew the particular lung and part of the lung ordinarily affected in phthisis.

Dr. Hughes wishes strongly to impress upon the minds of young auscultators the fact, that cases of phthisis are sometimes met with, though the number is comparatively small, in which tubercles appear to be simultaneously deposited in all parts of both lungs. Dr. Hughes again controverts "the assertion of a learned lecturer, that till dulness on percussion can be discovered, we are compelled to trust entirely to the general symptoms for the diagnosis of phthisis."

"Whatever may be the explanation of the fact, from repeated observation derived from a pretty extensive experience in the exploration of the thoracic viscera, in the practice of a large hospital for fifteen years, I am convinced that the lungs may be thickly sprinkled with tubercles, and yet yield a perfectly natural resonance upon percussion."

This certainly agrees with our own experience.

The disease is generally acute, and mostly occurs in young persons, especially females, with a strong predisposition to disease.

To proceed to the Table. It includes 250 cases, of which 175 were males, and 75 females. Of these there were 203; of which, 138 were men, and 65 women, whose chests were explored only during life. The remaining 48, of which 37 were men, and 11 women, were examined after death. A great proportion of the individuals whose names have been recorded among the living are now dead; but, either from permission not being obtained; from their leaving the hospital; or from other causes, the opportunity of making an inspection of their bodies was not afforded.

*Is one lung more obnoxious than the other to the deposition of tubercles?* The numbers, in 250 cases, stand thus:—

| | | Cases. | per cent. |
|---|---|---|---|
| The left side was chiefly diseased in | | 116 | 46 |
| The right ditto ditto | | 89 | 36 |
| The more diseased side was doubtful in | | 45 | 18 |
| Of the 116 cases on the left side, there were | males | 76 | 43 |
| | females | 40 | 53 |
| Of the 89 cases on the right side, there were | males | 66 | 38 |
| | females | 23 | 30 |
| Of the 45 cases in which the side was doubtful, there were | males | 33 | 19 |
| | females | 12 | 16 |

Of the 48 cases examined after death, of which 11 only were females, and 37 were males,

| | males. | females. |
|---|---|---|
| Tubercles were confined to the left lung in | 3 | 1 |
| right lung in | 1 | 0 |

The balance leans, though not very heavily, to the *left* side.

*In what proportion of cases are tubercles first deposited in the upper part of the lung?*—Of the two hundred and fifty cases, the upper lobe of one or both lungs was solely or principally diseased *in two hundred and thirty-seven*, or *ninety-five per cent.* Of the thirteen remaining cases, of which eleven occurred in males and only two in females, there were nine, or three and three-fifths per cent. of the whole number, in which both lungs were universally and uniformly diseased. Of these, eight were males, and one was a female. Of the remaining four cases, the upper lobe in three was at least equally affected with other parts. Dr. Hughes does not profess to say on what this remarkable tendency of the upper parts of the lungs to disease depends.

*At what age is phthisis most frequently fatal?*—Of persons who have died or were likely soon to do so, there were—

| | Males. | per cent. | Females. | per cent. | Total. | per cent. |
|---|---|---|---|---|---|---|
| Under the age of 20.. .. | 14 | 8 | 5 | 7 | 19 | 8 |
| Aged 20, and under 30 .. | 70 | 41 | 37 | 49 | 107 | 43 |
| Aged 30, and under 40 .. | 45 | 26 | 21 | 27 | 66 | 26 |
| Aged 40, and under 50 .. | 39 | 23 | 9 | 12 | 48 | 19 |
| Aged 50 and upwards.. .. | 7 | 4 | 3 | 4 | 10 | 4 |
| Total Males.. | 175 | Total Fem. | 75 | Grand Total | 250 | |

Dr. Hughes concludes with these conclusions: some of which may be dubious.

1. Tubercles may be simultaneously deposited throughout both lungs, and may then present no other physical signs than those of bronchitis.
2. This diffused form of phthisis is not necessarily acute and confined to young persons.
3. This form of the complaint is, comparatively, very rare.
4. Little difference exists in the liability of the two lungs to tubercular disease, but the left is rather more frequently first affected than the right.
5. In a considerable proportion of cases (18 per cent.) the disease advances equally on both sides.
6. Tubercles are very rarely confined to one.
7. In about ninety-five of every hundred cases of phthisis, tubercles are first deposited in the upper lobes of one or both lungs.
8. When the upper lobes are not primarily or principally diseased, they are almost always affected equally with other parts of the lungs.
9. The cause of this habit of tubercles is yet to be discovered.
10. Phthisis is most fatal to adults between the ages of 20 and 30; then, between 30 and 40; and then between 40 and 50.
11. Below the age of 20 and above 50, phthisis is, in adults, not frequently, but nearly equally, fatal.
12. Fewer females than males affected with phthisis attain the age of 40.

An interesting paper.

### VII.—On the Proceeding to be adopted in a Case of Injured Intestine, from a Blow upon a Hernial Sac. By C. Aston Key.

Mr. Key's attention has been directed to the treatment of these severe injuries by three fatal and two successful cases.

The accident usually occurs, either by a direct blow upon the lower part of the abdomen, or by the person being forced against some unyielding body, by which the truss is pushed aside, and at the same instant the intestine, descending into the sac, receives the full force of the collision. When the hernia is not supported by a truss, its descent takes place, and the blow is directly received upon the intestine. Sometimes the patient is unconscious of the existence of a hernia; and the symptoms that accrue, may be erroneously referred to an injury within the abdomen, or to the testicle and cord.

The injury to the intestine will vary according to the violence of the blow. The contusion may be insufficient to burst the bowel, or to occasion such a lesion of tissue as shall end in gangrene, its effects being only inflammation of the coats of the intestine; or the violence may be such as at once to rupture the intestine; or, failing to rupture the bowel, the contusion may be so severe as to be followed by sloughing and escape of fæces.

1. The mildest form of injury which a blow inflicts on a hernia is analogous to the contusion of other soft parts. The smaller vessels of the mucous and other tissues being ruptured, pour their contents into the reticular membrane, and thus gorge it with extravasated fluids. Such contusions, it is probable, are not followed by any serious consequences; nor will there be any material symptom beyond a certain degree of inaction in the muscular coat of the bowel, giving rise to temporary constipation.

"The two first indications that immediately force themselves on the surgeon's attention, are, the necessity of returning the contents of the hernial sac, and obtaining free evacuations from the bowels. To the former of these proceedings there can be no objection, as the vitality of the bowel is scarcely endangered: and if it were left in the sac, adhesion might form between the injured bowel and peritoneum, that would afterwards interfere with its return into the abdomen. The administration of purgatives ought to be wholly abstained from, notwithstanding the confined state of bowels usually consequent upon an accident of this nature. A bruised bowel is placed by nature in a state of rest: the exhaustion of the nervous energy of the part diminishes in the muscular tissue the disposition to contract. Such inactivity of the bowel should be encouraged, and not thwarted by irritating purgatives. The safety of the bowel depends on the non-occurrence of inflammation; but if, by undue interference, the bruised structure is hurried into a state of inflammation, sloughing or ulceration will probably be the result. Beyond an occasional enema, to unload the larger intestines, nothing need be done. Opium may be required, if pain come on, indicating peritonitis; and if joined with calomel, care should be taken that the action of the former should preponderate, in order to prevent the probability of stimulating the bowel. Food should also be given in the smallest quantity, and in a fluid form, that little or no feculent residue may remain, to oppress the part. In this respect, nature is our guide: vomiting, which usually ensues immediately after the accident, empties the upper part of the canal; and the little desire that the patient feels for food, prevents, if nature be allowed her own way, any chance of repletion. Thus the part is placed in a state of repose; and the circulation soon regaining its healthy condition, the functions of the intestine are restored."

Mr. Key adverts to the question of administering purgatives after the *operation* for hernia. He is decidedly opposed to the practice. We must confess that we are inclined to agree with him.

2. If the contusion, continues Mr. Key, be so severe as to destroy the vitality of the bowel without rupturing it, the condition of the patient, both immediately after the accident and for several days subsequently, sufficiently attests the severity of the lesion which the part has sustained. The hernial sac is usually found filled with the injured bowel; but the absence of distention serves to distinguish it from a state of strangulation. The integuments appear to be bruised, though sometimes but slightly. The part is very tender when handled, but feels soft and pliant; and very moderate pressure is sufficient to reduce the contents of the sac.

The shock which the nervous system receives, is followed by a feebleness of the circulation, a corresponding pallor of the whole surface, and a sense of syncope. This condition is however only transient: re-action almost immediately ensues; the patient passing from the state of collapse, and gradually rising into a state of inflammatory excitement, as the injured bowel becomes the seat of more or less inflammation.

The speedy recovery of the patient from a state of collapse quickly dissipates the suspicion of a rupture of the intestine; and the surgeon usually endeavours to replace the contents of the hernial sac as soon as re-action takes place. To this proceeding there is no objection, if it be done with gentleness. The danger of abdominal extravasation will not be increased by replacing the injured bowel at the neck of the sac: for should sloughing of its coats ensue, the slough may be walled in by adhesion of the surrounding peritoneum, and fecal extravasation be prevented; or, should this salutary process of adhesion fail to insulate the slough, the sac will receive the fecal matter, and quickly give intelligence of the impending mischief, by the tumefaction that will ensue within the scrotum.

The symptoms that arise in this state of things, in some points, resemble those of a strangulated bowel. The rejection of food from the stomach, the difficulty of obtaining stools, the tense and tender belly, and the swollen state of the scrotum, all lead to the impression that a portion of intestine has passed down through the rings, and has become incarcerated.

Mr. Key relates a case which seems to be in point. He also details the particulars of three others and concludes:—

It does not appear that persons are always aware of being the subjects of hernial protrusion: some, from carelessness, failing to notice the existence of a rupture; and others, from design, concealing their knowledge of the circumstance, when closely questioned as to enlargement at the rings. In two of the cases related, the patients did not seem to have been aware of any hernial swelling before receiving the blow. The history therefore of these cases, given by the patients, cannot be wholly relied upon. Their ignorance of the fact is not to be taken as evidence of a rupture not having existed previously to the blow: and it is of no little importance to establish the existence of a hernia; as without it, a rupture of a bowel is in the highest degree improbable, by a blow received upon the pelvis, or scrotum, or even upon the inguinal canal. Careful examination of the abdominal rings, and of the canal, can alone decide the absence or the presence of a hernia, and establish the probability, or otherwise, of the bowel being ruptured, so far as the existence of a hernia may favour it.

It may be urged, that the symptoms following upon a bruised intestine, or even upon a contusion of the testicle, may closely simulate those of a ruptured bowel. After these injuries, pains of a severe kind are felt about the scrotum and groin; the parts cannot endure rough handling, and vomiting sometimes follows. But these symptoms are transient; the shock passes away, and re-action ensues. The peculiar distress of countenance characteristic of ruptured bowel is wanting in the less severe injuries of these parts; and it is only the continued and increasing urgency of symptoms that fail to be relieved by the mild means resorted to, that should induce a surgeon to take the more serious view of the injury.

The period and manner in which the signs of lesion to the bowel shew themselves will serve sufficiently to point out to the surgeon the course which he is to adopt. The unnecessary exposure of a hernial sac and its contents is as much to be avoided, as delay is to be deprecated when extravasation of feculent matter is taking place. The former, however, is far the lesser evil; as experience shews that, in cases erroneously supposed to be strangulated hernia, the operation of opening the sac has been productive of no mischief. It is not likely that a surgeon, with ordinary discretion and knowledge, will be liable to mistake a mere external contusion, be it ever so severe, for a burst or injured bowel; nor will he be under any embarrassment how to proceed under the varieties of lesion to which the bowel is subject. The interval of ease that sometimes follows the blow, will not be taken as evidence of the bowel having received no lesion; for it seems, that until the peritoneum suffers from the presence of irritating matter, liquid or gaseous, upon its surface, the constitution does not take alarm. In the most severe injuries, when the rent in the bowel is extensive, and the fecal effusion almost instantaneous, the symptoms at once assume a character too marked to be mistaken; and the only mode of affording relief, namely, that of at once opening the hernial sac, is obviously pointed out. When the opening is so small as to prevent, for a time, any escape of the contents of the intestine, there is no necessity for any decisive step being taken, until called for by the unequivocal collapse and pain that attends extravasation. The interval of ease, as in the case of Jones, may lull for a time all suspicion as to the occurrence of rupture: but when extravasation begins, and not before, the surgeon's interference is called for; and no time should then be lost in affording an outlet to the offending fluids.

### VIII.—CASE OF IRIDEREMIA, OR ABSENCE OF IRIS; WITH OBSERVATIONS. By JOHN FREDERICK FRANCE.

*Case.*—Mary Hampton, aged 23, an out-patient at Guy's Hospital.

She is not aware of any other member of her family having been afflicted with complaint of the eyes; but has often heard from her parents (both now dead) and friends, that she was born with a defect of those organs. She herself has no recollection of ever having enjoyed more perfect powers of vision than at present; nor can she call to mind having suffered from any inflammation in the eyes, more important, or of longer duration than that which has accompanied other and more general catarrhal symptoms. During her infancy, and again six or seven years ago, her mother took her to the Moorfields' Infirmary; where the use of glasses was suggested—advice which she has not followed. The only occupation she has been fitted to pursue is that of household work.

*Present State.*—She is free, and ever has been, from any pain in the eyes, except that produced by exposure to strong light, which she cannot bear. Sunshine, in particular, is disagreeable to her, and produces profuse lachrymation: she therefore shuns it, preferring the dusk, when she can see better, and in comparative comfort. Objects are only distinctly seen when within the distance of a foot or two.

To the observer she presents the following appearances:—the eyelids are habitually more than half closed, and, from the permanence of this state producing an approach to entropion, and the shortness of the ciliæ, she bears, at first sight, the aspect of an individual who has been deprived altogether of eyelashes by disease. Such, however, is not the case; but, with the exception of slight catarrhal ophthalmia, the lids are healthy.

The globe of each eye is affected with an almost unceasing oscillatory movement in a horizontal direction; a symptom which, added to the spasmodic contraction of the orbicularis muscle, immediately excited on attempting to expose the eye to a good light, renders accurate observation of the condition of the deeper structures a matter of very considerable difficulty. The power of directing the eye towards an object, more particularly upwards or downwards, is much impaired.

Each cornea is partially clouded; the haze of the left occupying perhaps one-eighth of its superficies, and very slight: that of the right being more dense, nearly traversing the cornea horizontally, and rendering obscure about one-sixth of its surface. These are, of course, the relics of former inflammation.

The sclerotic coats are moderately healthy—perhaps rather more blueish than natural: their degree of tension is that of health. On inspecting the right eye very carefully, and looking above or beside the corneal nebula, there is observed a central opacity of the anterior capsule of the crystalline, about the size of a large pin's-head; and a similar spot is also to be seen on the posterior capsule; the lens remaining perfectly transparent. Thus a very satisfactory illustration is afforded of the size of the space between the cornea and capsule, and, again, between the front and back walls of the cavity within which the lens is contained. In the left eye, the centre of the anterior capsule, or more superficial part of the lens, is opaque; while the posterior, appearing as if corrugated, is opaque also in the centre, to about one-third of its extent; and shoots forwards, as it were, flakes of opacity into the lens on the nasal side.

With these exceptions, the entire space viewed through either cornea is of uniform brownish-black hue. The closest examination discloses no vestige of iris.

Owing to the obstacles opposed in this case to a scrutinizing inspection of the eye, which were before referred to—viz. the continued oscillation and unsteadiness of the globe, the intolerance of light, spasmodic contractions of the orbicularis, and, finally, the clouds obscuring in parts the surface of the cornea—I might still entertain some misgiving as to the real existence of this very rare malformation, and be inclined to ascribe to an unusual darkness of colour in the iris, and extreme dilatation of pupil or mydriasis, the non-appearance of the membrane in question. The result of the following mode of examination, however, superadded to the more direct ocular evidence, amounts to demonstration. If the observer, having gained sight of the posterior capsular cataract, and while watching it closely, gradually move his position, so as to look more and more obliquely thereupon through the cornea, he will find his view of the cataract not intercepted, until taken in so oblique a direction that the anterior edge of the sclerotic begins to intervene. Now, did ever so narrow a rudiment of iris exist, it would (leaving the ciliary ligament opposite to the junction of cornea and sclerotic to divide perpendicularly the aqueous chambers) hide the posterior capsule, viewed in the way described, before the point at which it, in this case, actually disappears.

### IX.—Case of Enormously-distended Gall-Bladder. Communicated by B. G. Babington, M.D. F.R.S.

Samuel Woods, aged 23, admitted into Job's Ward, Jan, 19, 1842; a tall man, with dark hair and eyes; by trade a plumber and glazier; states, that formerly he was of a very stout habit of body, and, with the exception of having had a fistula ten years ago, always enjoyed remarkably good health previously to his present illness. Thirteen months since, he began to suffer from swellings in his lower extremities, accompanied by great pain in his loins. These continued more or less till last April, when he first noticed beneath the margin of the ribs, on the right side, what he calls " a small ball," which, he states, was somewhat moveable, and gave him occasionally great pain; so that in the following month (May) he was compelled to leave off working altogether, partly from the pain which he suffered, and partly from progressively increasing weakness. Since that time the tumor has been gradually increasing to its present size, he has had an almost constant gnawing pain in his side, and has been rapidly losing flesh and strength.

*Present symptoms.*—His body is much emaciated; his face is exceedingly pale, waxy, and exsanguine: the conjunctivæ are of a pearly whiteness: the palpebræ dark-coloured. He complains of a gnawing pain in the abdomen, towards the lower part of the tumor. The legs are not œdematous at present, but he states that they become so when he moves about. His bowels are regular. The action of the heart, and the respiration are normal. Tongue clean. The right hypochondriac, and parts of the right lumbar, and umbilical regions, and of the scrobiculus cordis, are occupied by a large rounded tumor, the surface of which is quite smooth. It is moderately firm, and gives a very perceptible sense of fluctuation on percussion.

The pain augmented after his admission. On the 29th of January, an extremely fine exploring trochar and canula were passed into the tumor. No fluid escaped; but a fine probe, introduced through the canula, demonstrated that the tumor was full of fluid, as the probe passed without any obstruction for three or four inches beyond the canula's point. Although no fluid came through the canula, yet, when it was withdrawn, on passing the trochar again through it to clean it, a small plug of thick mucous fluid was forced out, which led to a speculation, whether the tumor might not be an enlargement of bowel, and this portion of thick mucus have proceeded from its lining.

The patient gradually declined. At 4 P. M. of the 11th of February, he complained of great pain over his whole body, which he compared to cramp. At 9 P. M. he expired.

*Dissection.*—The body, was emaciated, and the abdomen greenish. The serous cavities of the thorax seemed quite healthy. The peritoneum contained much muco-purulent secretion, which was evidently ropy, and mostly tinged with blood. About the spleen it was more watery; and above the liver, on the right side, very puriform. At this part, the diaphragm was extended, and thin, and coated (inferiorly) with thick, rugose, soft, adherent fibrin, which also covered parts of the liver. The omentum was contracted into a band resembling a large, pale, soft pancreas, and reaching to the right ilium. A great flaccid cyst adhered feebly but extensively to the anterior walls of the abdomen, and was in great part surrounded by hepatic substance; one acute margin of the liver being near the right iliac fossa. All the liver, to the left of the round ligament, was unaltered in structure: but it was thrust too much to the left. Much of the obtuse edge retained its form and place; but the remaining portion was pale and coarse in texture.

The cyst above-mentioned was more than half full of reddish, ropy, opaque secretion, about two large wash-hand basins full; with a copious sediment, as of a very puriform, semi-solid mucus, more or less in detached masses, variously tinted. The walls of the cyst were nearly a quarter of an inch thick; consisting of indurated liver and rather œdematous tissues, somewhat lacerable in a few points. One part nearly in front of the right kidney appeared to have given way before the inspection; and others gave way in the progress of separating adhesions which were of different degrees of firmness, but partial. The interior of the cyst presented many slight septa, in the form of flat folds, or sharp crescentic ridges, some four or five inches long and an inch in height. The lining had somewhat the appearance of a soft, rough cuticle. Parts of the wall involving the liver, seemed softened, and tending to suppuration. The fundus of the gall-bladder, expanded, thick, œdematous, and still reticular, formed the anterior parietes, and was about equal in superficial extent to a pint basin; and it was separated in part from the rest of the cyst by some of the crescentic septa above described.

Dr. Babington remarks:—"It did not appear in this case what had been the cause of obstruction to the cystic duct. No gall-stones were found; nor was there any trace of bile in the contents of the cyst. Whatever may have been its cause, I conceive that an inflammation had been set up in the gall-bladder: which led to the pouring forth of the mucus proper to the viscus, and ultimately, as suppuration advanced, of the fluid and semi-solid muco-purulent matter which has been already described."

We rather think there have been two or three cases recorded not very dissimilar to this. In one case a puncture was made by mistake into the distended gall-bladder. But in point of capacity this gall-bladder is probably unique.

### Report of Cases at the Chester General Infirmary, during the Years 1838, 1839, and 1840. By Thomas Beavill Peacock, Esq.

One principal point mooted in this Report is Fever.

The total number of cases of fever admitted in the years 1838, 1839, and 1840, were 53, 57, and 67, respectively, and the deaths 6, 9, and 9; the proportion thus afforded being 1 death in $7\frac{3}{8}$ patients, or 13.6 per cent. Of the cases admitted, 86 were males, 91 females; of the 24 fatal cases, 13 were males, and 11 females; the proportion of mortality was therefore 14.3 per cent. in males, and 15.1 in females. The average period of residence in the house of the cases cured was 25.4 days; the longest having remained 67, the shortest only 7 days. Of the cases proving fatal, the mean period of death was the nineteenth day from the commencement of the symptoms; the earliest having taken place on the sixth day, the patient being cut off by a suppression of urine coming on at the commencement of maculated fever during convalescence from acute rheumatism; the latest death was on the fifty-second day, the patient dying gradually exhausted by intestinal disease.

The ages of 157 patients admitted with fever were—

| | |
|---|---|
| Under 10 years of age | 2 |
| From 10 to 15 | 16 |
| .... 15 to 20 | 36 |
| .... 20 to 30 | 63 |
| .... 30 to 40 | 20 |
| .... 40 to 50 | 10 |
| .... 50 to 60 | 2 |
| .... 60 to 70 | 7 |
| Above 70 | 1 |
| The mean age | 25.7. |

The ages of those in whom the disease proved fatal were—

| | |
|---|---|
| From 15 to 20 | 2 |
| .... 20 to 25 | 7 |
| .... 25 to 30 | 4 |
| .... 30 to 40 | 3 |
| .... 40 to 50 | 3 |
| .... 60 to 70 | 4 |
| Above 70 | 1 |
| The mean age | 36.8. |

This table shews the greater fatality of fever at advanced than at early periods of life: under 40, the average mortality having been only 10.22 per cent.; above that age, 40 per cent.

*Eruption.*—The presence of the peculiar eruption, considered by many writers both abroad and at home as characteristic of the contagious form of typhus fever, has been very uncertain in its appearance in the fever of Chester. In 1838 and 1839 it occurred very rarely, and in 1840 only in 18 cases out of 53, though throughout the course of all the cases its appearance was attentively looked for. It has been supposed that the eruption appears very early and passes away; but of this Mr. Peacock could not satisfy himself.

Mr. Peacock observes, that there would seem to be a peculiar condition of the atmosphere by which the spread of all fevers attended with eruptions is facilitated, though the specific virus on which they may depend be different: thus, in 1839, small-pox was extremely prevalent in Chester, and proved fatal amongst children who had not undergone vaccination, and attacked also many who had the vaccine disease, though generally slightly. Scarcely had this ceased when scarlatina appeared, and prevailed extensively throughout 1840. Erysipelas also was of very frequent occurrence as an idiopathic disease, and complicated almost every case of wound or operation; and during this time the eruptive form of typhus, which for the previous year and a half had been seldom seen, occurred in a considerable number of cases. The circumstances under which it was met with were generally such as favoured the idea of its spreading by contagion. But the portion of the town from which the mass of the cases were admitted is extremely deficient in drainage, and the people are in a state of great wretchedness.

Of 17 cases in which a careful note was taken of the course of the eruption, in 4 only was its first appearance observed, all the others presenting it more or less copiously at the period of admission: in one of them it appeared on the third day, and continued till the seventh: in a second, on the fourth, and continued till the eighth; in a third, on the thirteenth, and remained till the twenty-first; and in the fourth, in which it also appeared on the thirteenth day, it continued till the period of death, on the sixteenth.

Of those cases in which it was present at the period of admission—

| | | | |
|---|---|---|---|
| In 3 | admitted on the 6th day | it continued till the | 11th, 12th, and 16th. |
| .. 3 | .. .. .. 7th | .. .. .. .. .. | 12th, 14th, and 17th. |
| .. 1 | .. .. .. 11th | .. .. .. .. .. | 19th. |

In 3 cases which proved fatal on the tenth day, it was found at the period of admission; on the fifth day, in 2 of the cases; and on the eighth, in 1. In 2 in which it was noticed on the sixth day at the time of admission, the period of decline was not observed.

The eruption appeared first on the lower part of the abdomen, and spread gradually over the chest, shoulders, and thighs, and in a favourable case generally declined a day or two before marked amendment in the symptoms occurred. In cases proving fatal in the early stages, it was observed, from being a delicate rose-coloured eruption, to become more purple, and to fade completely on pressure, at length assuming the form of petechiæ or vibices.

The youngest person on whom the eruption was detected was a child, aged 2½ years; six members of the same family having had fever in succession; the oldest was a man of 75.

In the cases attended with eruption, the fatality was much greater than in those without it; 6 out of the 18 occurring in 1841 having died, while of the remaining 35 cases only 3 terminated unfavourably. The convalescence of the cases cured was not, however, slower in the former than in the latter.

*Alkalescence of the Urine.*—The urine, it has been stated, becomes alkaline in the typhoid state; in most of the best marked cases its state was duly tested, and in none was it found so, though in two cases where typhoid symptoms supervened, on external injury, the urine was highly ammoniacal.

*Morbid Anatomy of Fever.*—There were 17 cases in which the head, and the same number in which the chest and abdomen were examined; in three instances the head only having been opened, while in other three it was not examined. The symptoms during life, in these cases, were observed and reported.

" In the case marked 6, in which the convolutions were found flattened, and the intergyral spaces obliterated, the fornix softened, &c. the patient was seized, during convalescence from a mild attack of fever, with coma, brought on by intense mental excitement. He became again sensible, but continued delirious at times till the period of his death, on the forty-ninth day. He died suddenly, and had no paralytic symptoms. In case 10, in which small patches of lymph existed on the membranes of the hemispheres, with slight effusion of fluid there, and distension of the ventricles, especially the left, with serum, the patient, after having complained of slight pain of head, intolerance of light, restlessness, &c. became suddenly comatose on the fourteenth day and was paralysed on the right side of the jaw. In case 8, the patient, whose brain exhibited great subarachnoid effusion, especially on the left side, with turgescence of the vessels of the pia mater, and roughness from small shreds of lymph, suffered at the time of admission from slight headache, then became torpid, then comatose, and died on the nineteenth day, having suffered for some hours before death from spasmodic twitching of the right arm. In cases 3, 9, 12, and 19, the subarachnoid effusion was great, and the convolutions small and widely separated. In one only was the brain unusually vascular, and in one the pia mater was turgid with blood, and rough to the touch. In one the brain was decidedly pale. These cases were characterised by the usual symptoms,—restlessness, suffused eyes, slight delirium, stupor, subsultus tendinum, and finally coma, with involuntary discharges; in all, retention of urine, with partial suppression, came on a day or two before death. In case 16 the symptoms in the early stages were those of high cerebral excitement, incessant restlessness, and delirium, rendering it difficult to keep in bed, yet with sensibility when spoken to. These gradually gave place to low muttering delirium, picking at the bed-clothes, and stupor. Here a great amount of effusion existed under the arachnoid and in the ventricles, and the cerebral vessels were much engorged. In cases 2, 4, 13, 14, 15, and 20, the disease was characterised by the usual nervous tremors of the hands, alteration of voice, deafness, stupor, but sensibility when spoken to, some delirium, &c. In these, effusion of serum was the only appearance which could be regarded as morbid in all but two, in one of which the red dots were more than usually numerous, and in the other the membranes were tinged with blood. In cases 5, 7, and 11, no morbid appearances existed to any extent in the brain, yet in one of them delirium was at first high and incessant, and continued to recur at intervals throughout the disease, and in the others the symptoms were similar to those last referred to.

If, then, in the above table, we except the three first cases, in which the marks of inflammatory action having been present in the brain and membranes were detected after death, and in which the symptoms corresponded during life, as instances rather of subacute inflammatory disease, we shall find that, of the remaining 14 cases, in 3 no appreciable disease was detected, and in 11 others the only morbid condition was effused serum on the membranes or in the ventricles, combined in 3 cases with turgescence of the vessels, and in 2 with increase of red dots. In several of these cases it is also stated that the convolutions were small, and widely separate, and the pia mater readily separable from the convolutions—circumstances which, taken in connexion with the age of the subjects and the well-known fact that few brains of elderly persons are examined in which serous effusion is not found to a greater or less extent, combine to show how little stress can be placed on these appearances, as explaining the symptoms present during life. The fluidity of the blood in most patients dying of fever, and the position which they assume in the last stage, will also account for the increase of red dots at the back of the brain, and in part for the engorgement of the posterior parts of the lungs, met with so often in their examination."

*State of the Glandulæ Aggregatæ.*—The peculiar disease of the glands of Peyer, considered by some French writers as constituting the proximate cause of the typhoid fever of the Continent, and on the presence or absence of which so much stress has been laid in the much agitated question of its identity with the fever of this country, occurred in an unusual proportion of the 17 cases examined. In seven of these ulceration was found either in the ileum, cœcum, or colon; in an eighth the follicles were enlarged and distended as if with solid matter, and a ninth also presented them elevated above the surrounding mucous membrane, but without any appearance of morbid deposit. In one of these cases, which proved fatal on the tenth day, the plates of Peyer were distinct, and elevated above the adjacent mucous membrane, and on each oval space there existed six or eight elevated pimples, which felt rough to the finger, and, examined with a magnifying-glass, weie found to present an open aperture at the apex, and to contain a yellowish-looking matter: considerable vascularity existed in the mucous membrane around. In another, in which death took place on the thirteenth day, it is stated that the plates were very distinct, elevated above the surrounding mucous membrane, and presenting numerous small openings on their surfaces, with larger ulcers here and there, near the valve of the cœcum. In a third, proving fatal on the sixteenth day, the plates were large, oval, and studded with openings along a considerable extent of the ileum, and near the cœcum the mucous membrane covering the whole plate was either ulcerated or so soft as readily to pull off. The vascularity in this case was distinctly greater around the plates than in other portions of the canal. The follicles did not present any appearance of solid matter in them. In four other cases the appearance of the follicles was entirely gone, and large ulcers with elevated and thickened edges and irregular surfaces occupied their places. These ulcers generally commenced a foot or two from the ileo-cæcal valve, and extended to the ascending colon. In some instances however they commenced higher up, and in one case were confined to the colon, in the whole extent of which they were found. In the small intestines they took the shape and direction of the plates, being oval, with their long diameter in the course of the canal. In the cœcum however they were extremely irregular in shape, and in the colon extended across the gut in the course of the folds of mucous membrane, forming in some cases almost entire rings. In the eighth case, the patient dying on the fifty-second day, the small intestines were studded with ulcers about the size of a sixpence, from the commencement of the jejunum to the lower portion of the colon. Their margins were depressed, and the surface smooth, and evidently in progress of healing. In several places the ulcers had perforated the intestinal tunics, and effusion of the contents of the canal into the abdomen had only been prevented by adhesions to the omentum or other portions of the viscera.

It appears from the reports of these cases during life that in one in which the plates were distinct, and had a few elevated pimples on them, with open apices, diarrhœa was present when the patient was admitted, on the seventh, and continued till his death, on the tenth day. In the next, in which the patient died on the thirteenth day, and the patches were found distinct, but with few ulcers, tenderness of the abdomen and tympanitic distension were present from the commencement of attack; and for the last three or four days three or four stools occurred daily. In the case proving fatal on the sixteenth day the symptoms were those of general prostration and some tenderness of abdomen, but evacuations were procured only by the exhibition of medicine, till the last day, when three stools took place spontaneously. In this case, it will be remembered, the ulceration was apparently commencing. In the others, which terminated between the seventeenth and twenty-second days, and in which ulcers occupied a larger or a smaller portion of the intestinal canal, diarrhœa existed for several days before death, and the indications of abdominal disease were present from the first. In one of these, a copious discharge of blood took place from the bowels on the fourteenth day; and in the one in which the ulcers had only been prevented perforating the peritonæal cavity by adhesions to the parts around, the symptoms of peritonæal inflammation took place on the ninth day. The case which proved fatal on the fifty-second day, and exhibited extensive intestinal disease in progress of cure, was characterized by repeated attacks of diarrhœa throughout its course.

Of the cases attended with the follicular disease, the average period of death was the twenty-second day; of those in which it did not exist, the eighteenth.

The same uncertainty which attended the appearance of the roseolous eruption in the cases admitted, was found to obtain in those in which the follicular ulceration was detected after death. In three of these cases the eruption was met with, and in a fourth an eruption made its appearance on the abdomen on the fourteenth day; but as it occurred at so late a period, and did not entirely fade upon pressure, it was regarded as petechial. In two cases, in one of which the roseolous eruption attended the early stage, numerous miliary vesicles appeared on the abdomen on the eleventh and eighteenth days.

The morbid appearances detected in the abdomen, independent of the disease of the intestinal canal, were, in several cases, a softened state of the spleen ; in one, a deeply congested condition of the mucous lining of the pylorus and duodenum; in one, grumous blood effused in the iliac space; and an engorged state of the kidneys in three out of the four cases in which retention of urine existed, one of which presented an aggravated example of the granular degeneration. In the thorax, the mucous lining of the bronchi was often more injected than usual, and the lungs, especially at the posterior portions, engorged, more particularly where the agony had been much prolonged. The larger vessels generally contained partially coagulated blood, and the heart was frequently more or less flaccid.

Mr. Peacock hazards the conjecture that:—perhaps much of the difficulty which attends this subject, founded on the frequent occurrence of the ulceration in the fever of certain localities, and its almost invariable absence in that of others, may in some degree depend on the comparative periods at which the cases ordinarily prove fatal. Thus, in the cases recorded in this report, the average period of death was the 20½th day; and here it occurred in eight out of seventeen cases. In forty-seven dissections taking place at the Royal Infirmary of Edinburgh, and recorded by Dr. John Reid, the average period of death was the 12½th day; and here only two cases occurred. When fever proves fatal at so early a period, it is probably by the disorder of the nervous system, the secondary lesions not having had time to develope themselves.

*Scarlatina—Secondary Deposits.*—Of three fatal cases of scarlatina, in one, death took place on the first day of illness, and of course before any eruption had appeared on the skin. The patient, a boy of nine years of age, was seized in the morning, though he had felt poorly a little before, with pain of head, suffused eyes, stupor, and sore throat, and extreme prostration of strength : he died in the course of the evening. The brain was greatly engorged with blood, but presented no effusion of serum; and the lungs, more especially the left, were excessively congested at the lower and posterior part; they felt solid, and did not crepitate. Several members of the family were labouring under scarlatina at the time.

The second case was that of a girl of fourteen, who passed favourably through a mild attack of scarlet fever, with affection of the throat, till the fourteenth day, when the febrile symptoms underwent great increase, attended with pain and tenderness of the knee, wrist, and elbow joints; and she died in two days. Copious effusion of pus was found to have taken place into all these joints.

The third case, that of a boy seven years of age, was fatal from effusion into the sac of the pleura, coming on suddenly after an attack of scarlatina, by which he had suffered so little as not to be confined to bed; about three pints of fluid existed in the two sides of the chest, the heart was large for the subject, and the kidneys much engorged; the other organs very pallid.

It must, we think, be apparent that these latter were cases of genuine secondary inflammations identical in character with those which follow accidents or operations. Now, we do not see how the theory of the resorption of matter will explain cases of this description, and, if it will not, we must hesitate to apply it unreservedly to others in which it may appear more appropriate.

*Congenital Atrophy of one side of the Brain.*—This was noticed in a case of phthisis. The weight of the left side of the brain was only twelve ounces and two drachms, troy, while the weight of the right hemisphere was seventeen ounces and seven drachms; the whole brain weighed thirty-six ounces and six drachms. The atrophy implicated chiefly, but not entirely, the superior portions of the anterior and middle lobes, the parts containing the ventricles being nearly of the same size on both sides: the anterior lobe, measured along the fissura Sylvii, was on the left side one inch and seventh-tenths, on the right two inches and nine-tenths. The membranes of the brain were extensively elevated by fluid, and the diploe remarkably thick, so that the inequality was not very conspicuous externally. This case was that of a nearly lunatic female, thirty-five years of age; the right leg was shorter and smaller than the left, so that she walked on the ball of the great toe, and the arm was contracted. Mr. Peacock has since seen a case in which, from the similarity of the deformity and flattening of the opposite side of the skull, he has no doubt a similar atrophy of the brain existed.

*Fracture of the Cranium.*—Of the four cases in which fracture of the bones of the skull occurred, in two the fissures extended across the petrous portions of the temporal bones on both sides, in one it took the same course on the right, and in the fourth on the left side. In one, a boy, who had been thrown from a horse, and dragged a considerable distance along the pavement, by his foot catching in the stirrup, the sphenoid bone was also found loosened from its connexions. In two of these cases the lateral sinuses were lacerated on both sides, and had led to very extensive extravasation of blood; and in another the same vessel was torn on the left side, a large opening existed into the longitudinal sinus at its point of bifurcation, and the middle cerebral artery was ruptured. In all the brain was extremely disorganized. In two cases bleeding from the ear occurred after the accident. In one of these a small portion of bone was found entirely separated, and raised up immediately over the internal ear, so as to allow fluid freely to pass through it; in the other the fissure was wide apart, and in this instance serum also escaped during life from the ear, and the collapsed and loose condition of the arachnoid, presented on examination after death, showed that it had escaped from beneath that membrane. One of these cases, the last, died about an hour and a half after admission, the patient having been sensible after recovering from the shock of the accident till immediately before his death. Two others lived eight and twelve hours respectively, and were insensible throughout. The fourth rallied from the first effect of the injury, so as to become partially sensible, and survived thirty-eight hours; he was however perfectly deaf, and on examination the auditory nerves on both sides were found torn through. Two of these cases were the effect of direct violence; the others of falls on the vertex.

*Mortality of Operations.*—Twenty-one capital amputations were performed; thirteen were of the thigh, of which four were performed for accidents; two on the immediate receipt of the injury, of which one recovered; and two were secondary, and took place on the sixteenth day after the accident: both of these proved fatal. Of the nine cases which were performed for disease, two died.

Of the leg, six amputations are recorded: of these, four were for accidents, three of which proved fatal, being performed soon after the injury; the fourth, also unsuccessful, took place on the seventeenth day. The two cases where the operation was undertaken for disease, both recovered.

Of the arm, two amputations occurred: one primary, one secondary, and both were successful.

The periods of death were—

| | | | |
|---|---|---|---|
| 2 within twenty-four hours after the operation, | | | |
| 1 | on the 8th day. | 1 | on the 17th day. |
| 1 | .... 11th day. | 1 | .... 28th day. |
| 1 | .... 14th day. | 1 | .... 30th day. |

In one of the fatal cases the patient proceeded favourably till the end of the third week, the stump being then nearly healed. In one, secondary hæmorrhage came on on the twenty-eighth day, when the stump, with the exception of a small sinus over the bone, was healed; the femoral artery was tied, and the patient ultimately recovered. In four of the fatal cases a careful examination of the body was instituted: in two, purulent accumulations were found in the knee-joint of the amputated limbs; in a third there was combined with these a similar abscess in the elbow of the opposite arm; in the fourth, matter had formed near the trochanters and on the surface of the ilium. In two other cases of external injury, one a comminuted fracture of the leg, and the other a bruise of the knee, both followed by diffuse inflammation, purulent deposits were detected in the lungs.

A highly creditable Report.

---

### MEATH INFIRMARY.

### Malignant Disease of the Eyeball successfully removed by Extirpation. By Dr. Byron.*

Richard Donnelly, a labourer, aged 21, was admitted into the Meath Infirmary, on the 4th of March, 1827. His left eye was affected with cataract, and with what appeared to be amaurosis; the conjunctiva was extensively chemosed, and the eyeball somewhat enlarged; the inflammation and lachrymation inconsiderable. He complained of headache, his pulse was 60 and full, his general health good; he had had no vision in the eye for a year: the other symptoms were only of a week's standing, and had been produced by striking the eye against a stick.

Three weeks afterwards it was deemed expedient to perform the operation for staphyloma, which was done on the 27th of March.

The sclerotic and choroid coats were divided three lines behind the ciliary ligament, and a large segment made of the ball, which included within it the lens in an opaque state, with its capsule and the iris, there was considerable bleeding, and some coagulated blood found its way into the anterior cells of the vitreous humour. There was much pain after the operation. The wound discharged pus and bloody matter in moderate quantity, and for some days the swelling appeared to be gradually diminishing; about a month, however, after the operation, the report was, that the wound had filled up, and the eye was as large as ever, having an uniform fungous aspect.

Nine months afterwards, he again presented himself; the tumor had increased in size very considerably within the preceding three months, and was then as large as a middle-sized orange. Its circumference at the base, measured nine inches, in the centre seven inches and a half, and at the top six inches; the eyelids adhered to its surface through the medium of the conjunctiva, which was moveable upon it. The anterior surface of the tumor presented an irregular aspect, from the existence of three protuberances upon it, each about the size of a walnut. The most prominent was of a dark purple colour, and bled when touched; the others were less vivid. The tumor was firm, elastic, and in some places hard; it had discharged as much as a pint of blood at one time; the general health was somewhat impaired.

---

* Dublin Medical Press, April, 20, 1842.

The disease advanced with rapidity; in a fortnight's time the tumor had much increased in size, and had begun to ulcerate; the tendency to hæmorrhage appeared to have ceased; for the first time, severe pain was complained of in the tumor shooting to the back of the head. The pulse was 80, and the general health, though impaired, had not materially suffered.

The operation of extirpation of the globe was performed on the 28th of February, two years from the original commencement of the disease.

*Operation.*—An incision, an inch and a half in length, was made from the external commissure to the external angle of the orbit. An incision was next carried through the conjunctiva, along the margin of the under eyelid, from the inner to the outer angle, where it joined the first incision; a similar division of the conjunctiva was then made along the border of the upper eyelid.

The surface of the tumor was now completely exposed all round, as far as the margins of the orbit. The tendons of the superior and inferior oblique muscles were then divided, so as to allow the finger to be passed freely round the orbit in all directions. The remaining attachments of the tumor, which were extensive, were afterwards divided, and the diseased mass, which was of great size, its posterior half filling up the orbital cavity, was removed.

The os planum was perforated, the edges were rough. There was some hæmorrhage from the ophthalmic and infra-orbital arteries, which was stopped by filling the orbit with lint.

*Examination of the Tumor.*—There was no appearance whatever of the lachrymal gland, and scarcely a vestige of the healthy eye; in place of the eyeball was a fleshy firm mass, on the surface of which ran the muscles, which were considerably increased in size. Beneath these was a strong tendinous coat, apparently the sclerotic, extended and altered in its nature; on stripping this off, the surface of the tumor was exposed; the whole superficies presented numerous tubercles of various sizes, from the dimensions of a pea to that of a chesnut, the larger being of a blackish colour, the smaller of a light brown. Between these tubercles was interposed a white semi-cartilaginous substance; the larger projections, on being divided, gave exit to a matter resembling the pigmentum nigrum of the choroid coat, and were composed of a more dense structure of the same nature. The smaller tubercles presented an aspect of the same sort, but lighter in colour; some were firm to the touch, others soft, the largest one being generally softest. The optic nerve was sound, but narrowed near its junction with the eye.

As to the termination of the case, it progressed favourably to a satisfactory cure. On the 10th day all fever had disappeared, and on the 88th day, the wound was completely healed, and the appearance of the parts good. There was a slight natural secretion from some small portions of the conjunctiva, which had remained adherent to the tarsal cartilages. He was discharged *cured*, and continued well, and perfectly free from any return of his complaint, for nearly four years afterwards, when he was carried off by a *fever*.

---

### NEW YORK HOSPITAL.

ON POLYPOUS TUMORS OF THE NASAL FOSSÆ. By J. WATSON, M.D. Surgeon to the New York Hospital.*

Dr. Watson, in his able Memoir, arranges the various forms of Polypus in this manner:—1st, the soft or mucous polypus; 2nd, the polypus from hyper-

* Amer. Journ. Med. Sciences, April, 1842.

trophy, induration and infiltration of the mucous and sub-mucous tissues; 3d, the fleshy polypus or caruncular excrescence; 4th, the fibrous polypus; 5th, the gelatinous polypus; 6th, the carcinomatous polypus. There are also on record, cases of lipomatous tumors originating within the nostrils, and some allusions have been made to osseous polypi, and to others supposed to depend on a scorbutic, scrofulous or venereal affection of the general system.

*Seats of the Disease.*—A knowledge of the exact points within the nasal fossæ, and other cavities at which these several polypi are most frequently attached, and of the form and extent of such attachment, is of much practical importance. Sir Astley Cooper states, that he has never seen a polypus growing from the membrane covering the septum narium. Mr. Sharp long ago observed that "they all arise from the membrane spread upon the laminæ spongiosæ." Mr. S. Cooper and others place their point of attachment between the turbinated bones near the orifice of the maxillary sinus; and Sir Astley Cooper and Professor Chelius, upon that portion of the Schneiderian membrane which is situated on the same side with the turbinated bones. Others, especially among the other writers, admit that they may adhere at any point within the nasal fossæ.

The points of adhesion for the first two forms of polypi, are probably confined to the tissues lining the external and upper wall of the nostril, and the maxillary sinus. The seat of the third form is, perhaps, not so strictly limited, its most frequent attachment is near the external orifice of the nostril, at or below the turbinated bones. The fourth form is equally indefinite in its point of attachment. It is most frequently found near the posterior nares; it may arise in the posterior fauces, immediately behind the top of the septum, and probably from the septum itself. The fifth form, or the gelatinous polypus, as already shown, may arise in the maxillary sinus, and in its progress form adhesions within the sphenoidal and ethmoidal cells, along the spheno-maxillary fissure, and on the floor of the nostril. Finally, the carcinomatous polypus, though frequently arising on the nasal surfaces of the upper maxillary bone, is restricted to no definite point of attachment; it is rarely or never pedunculated, but in its progress it attacks every part alike. Besides the situations above specified, there are cases on record, showing that polypous tumors may originate in the frontal sinuses, on the inner surface of the nasal bones and ossa palati; the under surface of the os unguis; within the cells of the ethmoid and sphenoid bones; at the side of the pharynx, at the base of the tongue, and in the upper and anterior part of the œsophagus; not to mention various remote parts of the body.

*Diseases likely to be confounded with Polypus.*—"1st. Chronic Coryza, or inflammation and thickening of the pituitary membrane, and of the cellular tissue immediately beneath it. Several instances in which this disease has been confounded with, or mistaken for polypus, have fallen under my own observation; in some of which the polypus forceps had been employed, and the patients subjected to needless and severe laceration of the pituitary membrane.

2nd. Follicular ulceration of the mucous surfaces, an affection analogous to, or identical with the follicular disease which sometimes attacks the external surface of the nose, constituting the scrofulous form of noli me tangere. In one of the few examples of this, which have fallen under my own observation, the disease had at one period been supposed to depend upon a polypus.

3rd. Ozæna, whether the result of scrofula, carcinoma, or syphilis, especially in its early stages, prior to the exfoliation of the bones and cartilages, and before the fætor becomes so manifest, as it usually is in the advanced stages of this disease. In one of the cases already reported, before the tumor was detected in the nostril, the disease had been confounded with ozæna.

4th. An unusually large projection of the free border of the lower turbinated bone has, in more than one instance, been mistaken for polypus. The mistake is most likely to occur when this projecting edge is rendered still more prominent by thickening of its mucous envelope.

5th. An inordinate distortion of the septum, causing a projection into one of the nostrils, and obstructing the passage of air through it, has occasionally been mistaken for polypus. M. Velpeau refers to a case of this sort, in which a portion of the septum was mistaken for a polypus, and literally torn away by means of the forceps. I have, at present, under care, an hospital patient, in whom a similar mistake, on the part of the physician, resulted in ulceration of the septum, and final exfoliation of the vomer and part of the cartilaginous partition of the nostrils.

6th. A hernial protrusion of a part of the brain and of its envelopes, may descend into the nostril through an opening in the cribriform plate of the ethmoid bone, and then be mistaken for polypus. An instance of this sort is recorded, I think, by Cruveilhier.

7th. Foreign bodies introduced into the nostril. Boyer relates an instance, in which a pea thus introduced by an infant, was mistaken for a polypus. It had germinated and expanded its roots to the length of three and a quarter inches."

1. *Mucous Polypus.*—Dr. Watson thinks that the true cause of these tumors is the accumulation of mucus within the muciparous follicles, which may occur either from a change in the consistence of the mucus itself, or from obliteration or obstruction of the ducts. In this respect they are closely allied to the sebaceous and encysted tumors of the scalp and other parts of the body, and that are usually found immediately below the skin. Dr. Watson relates a case. The polypus, when removed, might have weighed two ounces; it was of a pale flesh colour, and lobulated. The lobules were bulging on the surface, they varied much in size, and was separated from one another by bands of tolerably firm cellular membrane. They were composed of semi-transparent sacks filled with fluid, and when punctured, gave issue to a quantity of mucus similar to the natural secretion of the nostrils.

2. *Polypus from Hypertrophy of the Mucous and Submucous Tissues.*—It occurs perhaps almost as frequently as the first; and the two forms are often seen together, as in case third. In the one, however, the tumor may acquire considerable size without producing either condensation or thickening of the mucous membrane; whilst in the other this condition appears to be the starting point of the disorder.

The whole of the pituitary membrane is sometimes found in a state of hypertrophy or thickening, the result either of local inflammation or of a pruriginous irritation or follicular disease of the integuments. This condition gives rise to most of the inconveniences of polypus nasi: but unless some portion of the mucous surface becomes more developed than the rest, so as to assume the character of a tumor, the disease is not to be considered under this head.

3. *Of the Fleshy Polypus.*—This is vascular, of a florid red colour, and though not painful except when irritated, it is possessed of a certain degree of sensibility; it is less disposed to assume a pedunculated attachment than any other benign form of polypus.

4. *Fibrous Polypus.*—Dr. Watson thinks that the fibrous tumor of the nostril is invariably attached by a pedicle, quite as firm and fibrous as the tumor itself. Such, however, is not always the fact in fibrous tumors of other parts of the body. These nasal tumors, as seen projecting into the pharynx, are usually pyriform, except when altered in shape by the pressure of surrounding parts. They are frequently solitary. They sometimes acquire an enormous size, encroach upon the parts surrounding them, produce great deformity of the head and face, and lead to extensive ulceration, repeated hæmorrhage, destruction of the bones, and even to fatal consequences. The older writers, aware of the formidable symptoms resulting from this tumor in its latter stages, were led to consider it malignant. According to M. Velpeau, it has its special origin in the fibrous tissue that invests the bones of the nasal fossæ, and that lies between the bones and the proper mucous tissue; whilst this latter tissue, according to the same author, is the special seat of the softer polypi.

5. *Gelatinous Polypus.*—What Dr. Watson means by this may be seen from the dissection of a case. "The morbid mass was found blocking up and distending the antrum, the parietes of which were burst asunder and excessively attenuated, being on the cheek not thicker than an egg-shell. The septum narium, and bony palate, were either absorbed or much distorted and attenuated. The mouth and pharynx were filled with the tumor, which also extended into the posterior part of the orbit, and through the spheno-maxillary fissure towards the left side of the head. It involved the ethmoid and sphenoid bones, the cells of which were filled with it; and extended through the latter bone to the base of the brain; but whether the upper plate of the sphenoid bone had been absorbed, or broken in the examination, could not be ascertained. The whole of the bones here, however, were softened and diseased, rather by inflammation or interstitial absorption, produced by pressure, than by degenerating into a substance similar to the tumor itself.

The disease appeared to have originated in the antrum, probably between the mucous membrane and the bone, and without involving the bone otherwise than by pressure, to have extended from this to the surrounding cavities. The morbid deposit was surrounded by a sort of imperfect capsule, with subdivisions of cellular tissue, some of these exceedingly delicate, and all of them filled with a gelatinous semi-fluid substance, of a transparent pale white or amber colour. At some points this matter was more like soft calf's-foot jelly, without any visible envelope; but here and there the tumor contained opaque grumous bloody deposits, and these were most abundant at the outer lobes of the tumor, just in front of and below the ear. The brain, except at the cella turcica, was unaffected."

We apprehend that, in this country, the term "gelatinous polypus" is commonly applied to what Dr. Watson describes as the "mucous." His gelatinous polypus seems to be malignant disease.

6. *External Polypus Nasi.*—By this Dr. Watson intends the lipoma or hypertrophy of the integument of the nose which sometimes gives an organ so prominent and jolly an appearance. It is doubtful, we think whether this ought to be ranked with polypi at all.

7. *Carcinomatous Polypus.*—Of this we need not speak.

Treatment.—*Mucous & Soft Polypi.*—Dr. Watson observes that the mucous polypus, and that depending on hypertrophy, induration, infiltration, &c. of the Schneiderian membrane, are most readily and effectually removed by the straight forceps. Their most common point of attachment is on the outer wall of the nostril. For the purpose of seizing them the blades of the instrument, slightly open, should be passed gently along the nostril until they reach the base of the tumor, and there cautiously closed upon it. After one or two slight movements to ascertain that the instrument is properly applied, and that none of the normal structures are embraced in it, the surgeon, holding the blades firmly together on the neck of the tumor, rotates the instrument upon its longitudinal axis until he is satisfied that all resistance is overcome, and that the morbid mass is twisted off; and then, without relaxing his grasp, he gradually withdraws the tumor from the nostril. This done, the cavity is again to be examined, and if any part of the tumor has been left in it, or if other tumors are discovered, the process is to be repeated until all obstruction is removed. The patient usually ascertains this fact of himself, by the instantaneous freedom he experiences in breathing. The curved forceps are applicable only to such tumors as are inaccessible to the straight instrument, or where the tumor is situated so far back as to be most easily removed through the mouth. When introduced in this way, care must be taken that the instrument do not injure the soft palate.

The treatment by puncture is in general to be looked upon as a substitute for the more efficient measures. In some cases, however, it may effect a permanent cure. It is best adapted to recent polypi, the lobes of which are few but large and unattended with thickening or induration of their investing membranes. In order to be effectual the punctures should be deep. In operating by puncture, we should first draw the tumor gently outward with the forceps, and then plunge the blade of a narrow straight bistoury through the body of the tumor toward its base, but not so far as to reach or injure the sound parts. The point of the instrument without completely withdrawing the blade, may then be made to pass in different directions through the morbid texture, or new punctures may be made at different points upon the surface. The mucus flowing from the punctures thus made very soon allows the tumor to collapse.

*Caruncular Excrescence.*—The caruncular excrescence and other vascular polypi not of a carcinomatous character, when small, may be effectually treated by a few applications of lunar caustic, or any other mild escharotic, care being taken not to injure the sound tissues, especially those near the upper edge of the ala, where the parietes of the nostril are very thin. When the tumor is of considerable size, and already pendulous, it may be removed with the scissors or bistoury, its base being subsequently touched with caustic. When the peduncle of a flabby tumor is too broad to allow at once of removal by incision, the safest and most expeditious plan will be, first to apply a ligature to the base, and afterwards to separate the neck or body of the tumor by an incision at a little distance beyond this. But if the tumor be not easily brought within the reach of the scissors or bistoury, after applying the ligature tightly *en masse* upon its base, it may be left to slough. In instances of this sort it may be necessary to tighten the ligature from day to day, as practised and recommended by Heister.

*Fibrous Polypus.*—The fibrous polypus may be attacked with the forceps, scissors, or bistoury; with the loop or ligature for evulsion; with the permanent ligature *en masse* for strangulation; or simply with the fingers of the surgeon.

When the neck of the tumor is narrow, readily reached, and of a texture that can be easily separated, it may be divided with the scissors. If less accessible, or of firmer texture, the forceps will prove the most efficacious instrument. In a case of this sort, after other means had failed, Dr. Physick succeeded in casting a tape around the base of the tumor and then forcibly tearing it from its attachment. The permanent ligature in these cases requires frequently to be tightened; it is tedious and annoying to the patient: is liable to failure, especially when the neck of the tumor is large and of the same fibrous character as the other parts of it. There are instances, however, in which the permanent ligature offers advantages over every other mode of treatment, and in some cases it is our only resource.

---

### LUNATIC ASYLUM OF ABERDEEN.

The Medical Report for the year 1840 is before us. We have omitted to notice it before. There are one or two points to which we may direct attention.

*Moral Insanity.*—The reporters, Drs. Macrobin and Jamieson, touch on this ticklish subject. The term moral insanity, say they, is employed to include those cases in which there is present no incoherence nor aberration of the *reasoning* powers, but merely such a degree of violent and uncontrollable passion, or of extravagant and whimsical conduct, as to render the individual dangerous, —ridiculous,—and incompetent to manage his own affairs. Though either a consequence or a precursor of some more decided modification, it is not unfrequently in itself a primary and persistent form of mental derangement. For as great eccentricity may exist, through all the customary vicissitudes and excitations of a long and active life, without being once overstretched into glaring and irrational incongruity; so may a state of Moral insanity continue, without assuming the more evident form of Intellectual disorder. Betwixt slight incapacity of control, and absolute confusion of ideas, the stages are ordinarily progressive and dependent; but it is quite in accordance with observation, that any one of these may acquire such a persistency, as at no time to show a tendency to merge into the still more mature modification which is its general consequent. Thus, though they have all and each a liability to progress,—eccentricity may never pass into Moral Insanity,—nor the latter into Mania,—nor Mania into Fatuity and Incoherence.

Until the existence of a moral perversion, as an independent form of Insanity be generally allowed, and its phenomena more distinctly understood, difference of opinion as to the responsibility and non-competency of individuals, will continue to arise between Medical Men, and Non-professional Juries, however unprejudiced and intelligent.

As we have given above a few of the common features of this species of derangement, we may add some of the symptoms which are premonitory of its approach. These frequently are, estrangement and perversion of the natural affections,—unusual obedience to sudden impulse,—great violence of passion,—extreme waywardness of disposition,—and restless activity of intellect. In every instance where in early life eccentricity exists, and still more especially when it is conjoined with hereditary predisposition to Insanity, all occasions of excitement should be carefully withdrawn, and habits of restraint and regularity unremittingly and judiciously enforced."

No doubt there is such a thing as *Moral Insanity*, and it would be well in many cases, for their families and the public, were these moral madmen shut up. But it is necessary for the interests of society, to look at questions of this description in the broadest point of view. If there be a practical as well as theoretical difficulty in determining on the amount of ordinary madness which should doom the lunatic to the asylum, this difficulty must be ten times greater in the case of moral madness. No broad and tangible barrier separates *that* from the usual eccentricities of human character, and it is perhaps questionable whether any man would bear a searching scrutiny into his *moral sanity*. The question is altogether one of plus or minus, and great discrimination as well as caution, are requisite to determine it in individual cases. Were the modern doctrine of moral insanity generally acted up to, what a scope there would be for oppression and abuse. The husband who desired to be quit of his wife, the wife who longed to get rid of her husband, might find the mad doctor a convenient ally, and the madhouse a convenient prison. A political opponent, an object of popular aversion might be immolated at the shrine of prejudice or passion. We had better bear the ills we have than hang over our heads so formidable a weapon as a statute of "constructive" madness of this sort.

*Hereditary Predisposition.*—Out of the seventy-three cases in the 5th table, thirty-eight could be distinctly traced either to a natural or an acquired constitutional predisposition; and out of a hundred and twenty-six cases admitted, during a period of two years, as shewn in the second of the preceding tabular statements, sixty-five had originated from a similar tendency.

*Intemperance* was the chief of the physical causes, but, say the Reporters, though intemperance is frequently an exciting cause, it is likewise often merely a symptom of previous mental disorder; and this is well verified in the instances of several individuals at present in the house, in the histories of whose cases, it is stated, that one of the first warnings appreciated by friends, was the exhibition of an unwonted predilection for ardent spirits.

*Mental Application.*—In only one case did excessive mental application appear to have been the sole cause, there being no ascertainable constitutional predisposition. Perhaps it may be doubted if this is by any means a common cause of insanity, for certainly the mind is much more liable to be over-excited and injured through the agency of the passions, affections, and other operations of the Will, than it ever is through any of the faculties of the Understanding. In almost all instances in which overstrained intellectual exertion is given as a cause, it will be found, that some one of the passions has been in a state of great and continued activity; the individual having, for example, been long agitated by some such feeling as anxiety, ambition, or a dread of disgrace. The physical excitement which commonly characterizes the incipient stage of mental derangement, is far more readily engendered by the emotions and passions, than it is by any of the intellectual operations of the mind.

*Average Period of Cure.*—Twenty-six cures have been effected in the course of the last twelve months,—of males and females the numbers being equal; fifteen of these were cases of Mania, and the remaining eleven all instances of Monomania. The majority in favour of the Maniacs will appear much more striking, when it is considered, that the Monomaniacs constitute by far the greater proportion of the admissions.

Of the seventy-three cases admitted, only forty-five were brought to the Asylum in a state affording a reasonable hope of cure; and of these, sixteen have already been discharged recovered, and six materially improved.

The average period which has been occupied in the cure of the foregoing twenty-six patients, is about twenty-nine weeks; sixteen weeks being the average in Mania, and forty-two in Monomania. The average duration of treatment in the male cases, is about thirty weeks; in the female, about twenty-seven. The average in male Maniacs is rather more than sixteen weeks, and in the female rather less; in male Monomaniacs, rather less than forty-five weeks, and in the female, rather more than thirty-eight.

Hence it may be inferred that the male are less readily cured than the female patients; and that a longer period is necessary for the treatment of Monomania, than is requisite for Maniacal cases,—or that, *cœteris paribus*, female Maniacs are the most favourable, and male Monomaniacs the least so, of our curable cases. It may likewise be taken, as a well established observation, that patients, in whose cases treatment by removal from friends, and confinement in an Asylum has been delayed, require in general a longer period for cure, than those in which such measures have been earlier adopted.

*Attendance on Divine Worship.*—In respect to divine worship on Sundays, the question of its advantage seems to be settled: its utility in all institutions where it has been introduced, being unanimously allowed. It is not only useful as a Sunday occupation,—and without it, Sunday would be the most hurtful day in the week to the insane,—but in many cases, we have no hesitation in saying that it is positively beneficial, both in advancing the cure, and in confirming the convalescence. In general, we allow all to attend who are out of bed, and not under restraint, if they have the sole qualification of being capable of remaining quiet during the time of the short services which are performed; and this we do without much respect to the nature of the delusion. At no other time, the Reporters protest, are the insane so like rational and accountable beings.

